# A Combination Therapy Using Electrical Stimulation and Adaptive, Conductive Hydrogels Loaded with Self‐Assembled Nanogels Incorporating Short Interfering RNA Promotes the Repair of Diabetic Chronic Wounds

**DOI:** 10.1002/advs.202201425

**Published:** 2022-09-05

**Authors:** Huan Lei, Daidi Fan

**Affiliations:** ^1^ Shaanxi Key Laboratory of Degradable Biomedical Materials Shaanxi R&D Center of Biomaterials and Fermentation Engineering Biotech. & Biomed. Research Institute Northwest University Taibai North Road 229 Xi'an Shaanxi 710069 China

**Keywords:** diabetic chronic wounds, hydrogel‐ES combination therapy, self‐assembled nanogels, siRNA delivery

## Abstract

In addition to oxidative stress and impaired angiogenesis, the overexpression of metalloproteinases (MMPs) and proinflammatory cytokines, which are promoted by hyperglycemia, causes chronic inflammation in diabetic wounds. Herein, TA‐siRNA nanogels are prepared for the first time on the basis of the self‐assembling interaction between tannic acid (TA) and short interfering RNA (siRNA). The efficient, biodegradable nanogels are cross‐linked with poly(vinyl alcohol) (PVA), human‐like collagen (HLC), TA, and borax to prepare adaptive, conductive PHTB (TA‐siRNA) hydrogels. In response to high levels of reactive oxygen species (ROS), the ROS‐responsive borate ester bonds in the hydrogels are oxidized and broken, and TA‐siRNA nanogels are released into cells to reduce the expression of the MMP‐9. Moreover, the TA and HLC promote collagen expression, reduce inflammation, and ROS level. It is found that electrical stimulation (ES) promotes the in vivo release of TA‐siRNA nanogels from PHTB (TA‐siRNA) hydrogels and endocytosis of the nanogels. The combination therapy using ES and PHTB (TA‐siRNA) hydrogels accelerates the healing of diabetic wounds by reducing the levels of ROS and MMP‐9 and promoting the polarization of macrophages, production of collagen, and angiogenesis. This study provides insights on the design of functional gene‐delivery and efficient therapeutic strategies to promote the repair of diabetic chronic wounds.

## Graphical abstract

The efficient, nontoxic, biodegradable self‐assembled tannic acid‐short interfering RNA (TA‐siRNA) nanogels could promote the effective cellular internalization of siRNA to reduce the expression of the target gene. Adaptive conductive hydrogels combined with electrical stimulation (ES) could promote the repair of diabetic chronic wounds by scavenging reactive oxygen species (ROS), metalloproteinases‐9 (MMP‐9), and proinflammatory factors and promoting macrophage polarization and vascularization.

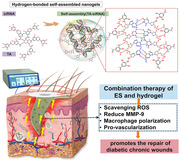

## 1 Introduction

The worldwide number of cases of diabetes mellitus (DM), which is one of the most common chronic diseases, is expected to exceed 590 million by 2035.^[^ ^1^ ^]^ DM is characterized by a persistently high level of blood glucose (i.e., hyperglycemia), and chronic wounds, which are complex and intractable complications, are expected to prevail among 19–34% of diabetic patients.^[^ ^2^ ^]^ Hyperglycemia is responsible for the difficulty to repair the chronic wounds: it impairs the formation of new blood vessels and induces the production of free radicals, overexpression of metalloproteinases (MMPs), and high expression of proinflammatory cytokines. An abnormal glucolipid metabolism and oxygen deficit, both of which are due to DM, create a hypoxic environment with a high degree of oxidative stress.^[^ ^3^ ^]^ An excess amount of reactive oxygen species (ROS) induces a strong inflammatory response and inhibits macrophage functions, blood‐vessel formation, and cell regeneration, thus, hinders the repair of wounds.^[^ ^4^ ^]^ On the other hand, excessive proinflammatory factors increase the levels of MMPs and promote the degradation of proteins, thus, impair cellular structures and inhibit extracellular–matrix (ECM) formation.^[^ ^5^ ^]^ Therefore, researchers have undertaken considerable efforts to improve the existing therapeutic pathways and/or develop new effective therapies to address the complex challenges associated with DM.

Active ingredients (e.g., antibiotics and growth factors) may reduce chronic inflammation and promote wound repair.^[^ ^6^ ^]^ However, they have some setbacks such as drug resistance and high costs.^[^ ^7^ ^]^ Compared to the active ingredients, short interfering RNAs (siRNAs), which are expected to be next‐generation drugs, are cost effective and have several advantages such as unrestricted applications to any target proteins, specificity to target mRNAs, and simplicity of the manufacturing process.^[^ ^8^ ^]^ However, the use of siRNAs for effective treatments is limited by the lack of effective delivery systems.^[^ ^9^ ^]^ On their way to target tissues and cells, siRNA carriers may be removed by the endothelial system or contaminated with proteins. Therefore, the design of carriers that can facilitate the passive delivery of siRNAs to target tissues and internalization of the siRNAs by target cells is an important direction in the research on the delivery of siRNAs. Nanogels and hydrogels have been developed as the carriers for the transfection of siRNAs into cells. By using hydrogels and nanogels, researchers have delivered siRNAs to silence target genes in the kidneys, skin, and ovary.^[^ ^10^ ^]^

In our previous work,^[^ ^11^ ^]^ we used polyvinyl alcohol (PVA), human‐like collagen (HLC), tannic acid (TA), and borax to prepare PHTB hydrogels that are multifunctional, adaptive, and conductive. HLC is a water‐soluble protein expressed by *Escherichia  coli* with human‐derived genes, and it has good cell proliferation and collagen expression properties.^[^ ^12^ ^]^ On the other hand, TA is a natural polyphenol with good anti‐inflammatory and antioxidant properties. The hydrogels are cross‐linked by dynamic borate ester bonds and hydrogen bonds. Due to their adaptive and conductive properties, the hydrogels are able to fill deep wounds and facilitate intercellular signaling in tissues. The hydrogels have hemostatic, anti‐inflammatory, antibacterial, and pro‐cell proliferation properties and exhibit therapeutic effects throughout the phases of the healing of acute wounds. We used a combination of PHTB hydrogels and electrical stimulation (ES) to treat acute wounds. The hydrogels improve current transmission, and the ES promotes cell migration, angiogenesis, cell proliferation, and collagen deposition. Thus, the combination therapy accelerates wound healing.

**Scheme 1:**
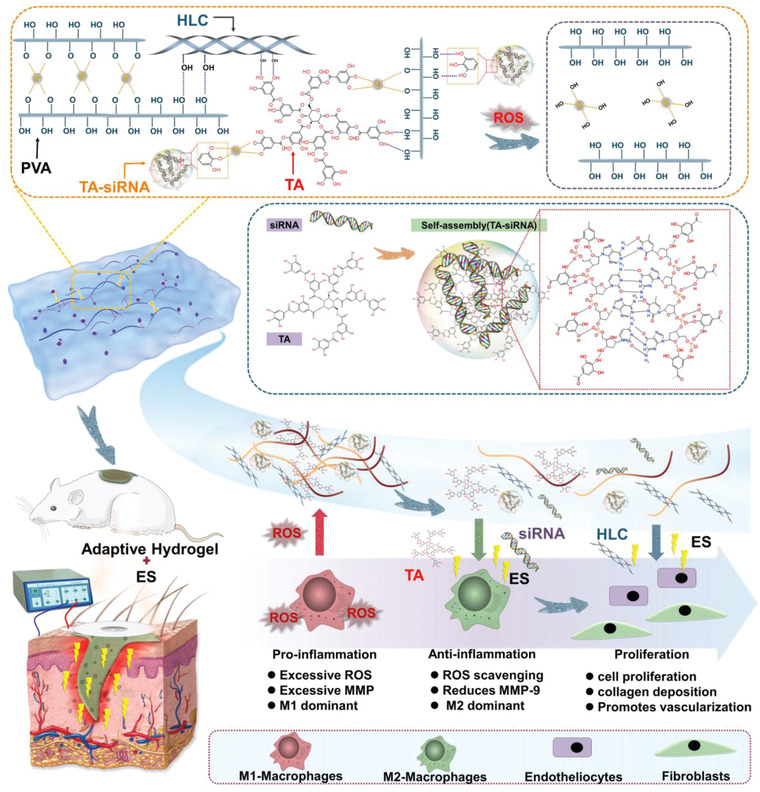
The repair of diabetic chronic wounds using the combination of ES therapy and adaptive, conductive PHTB(TA‐siRNA) hydrogels.

In comparison with acute wounds, chronic diabetic wounds have more complex pathological features. Therefore, in this work, we improved the therapeutic efficacy of PHTB hydrogels to treat chronic diabetic wounds. We focused on the following therapeutic aspects of the hydrogels: antioxidant (i.e., ROS scavenging), anti‐inflammatory, pro‐angiogenic, and MMP‐inhibition properties. We constructed TA‐siRNA nanogels (**Scheme** **1**) by using a self‐assembly strategy that allows TA to polymerize and cross‐link with siRNA to form homogeneous nanogels. The “green” TA‐siRNA nanogels, which are nontoxic and biodegradable, exhibited high gene‐silencing efficiencies in vitro and in vivo. The nanogels were introduced into adaptive, conductive PHTB hydrogels, and the resulting adaptive, conductive PHTB (TA‐siRNA) hydrogels were used in combination with ES therapy to repair diabetic chronic wounds. We focused on the following aspects: i) the cellular internalization and gene‐silencing efficiency of the TA‐siRNA nanogels and effect of ES therapy on the cellular internalization of the nanogels; ii) the ability of the borate ester bonds in PHTB (TA‐siRNA) hydrogels to respond to ROS levels in diabetic chronic wounds and effectiveness of the hydrogels to scavenge ROS; iii) the mechanisms for the repair of diabetic chronic wounds using the combination of ES therapy and PHTB(TA‐siRNA) hydrogels, including inhibition of inflammation, scavenging of ROS and MMP‐9, polarization of macrophages, formation of blood vessels, and production of collagen. Our previous study focused on the efficacy of PHTB hydrogels to repair acute wounds and ability of the hydrogels to improve current transmission and enhance the efficacy of ES therapy. On the other hand, in this study, we investigated the gene‐delivery efficacy of TA‐siRNA nanogels and ability of PHTB(TA‐siRNA) hydrogels to respond to ROS levels and scavenge ROS. Moreover, we studied the effect of ES therapy on the release of TA‐siRNA nanogels from PHTB(TA‐siRNA) hydrogels and cellular internalization of the nanogels. In addition, we investigated the mechanism for the repair of chronic wounds by using the combination of PHTB(TA‐siRNA) hydrogels and ES therapy.

## 2 Results and Discussion

### 2.1 Construction and Cellular Internalization of TA‐siRNA Nanogels

**Figure 1:**
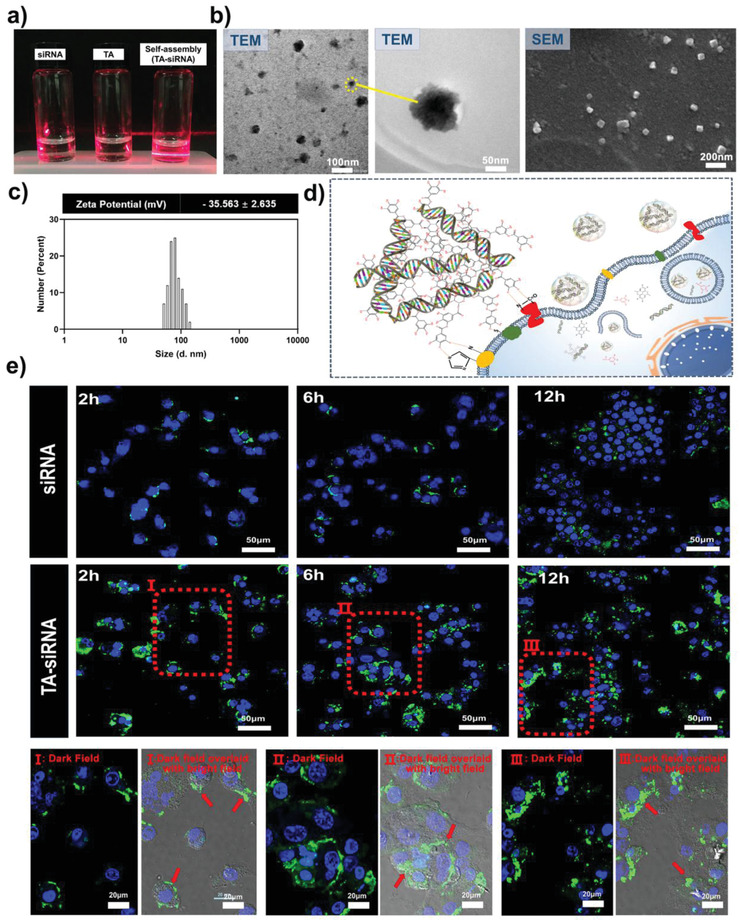
a) The Tyndall effect and b) transmission electron microscopy (TEM) and scanning electron microscopy (SEM) images of TA‐siRNA nanogels. The scale bar of TEM represents 50 nm and 100 nm scales, respectively. The scale bar of SEM represents a 200 nm scale. c) The size distribution and zeta potential of the nanogels (*n* = 3). d) The mechanism for the adhesion of the nanogels to cells. e) Confocal microscopy images of macrophages after the cellular uptake of TA‐siRNA nanogels. The scale bar represents a 50 µm scale. I, II, and III are the high‐magnification confocal microscopy images of TA‐siRNA internalization at 2, 6, and 12 h, respectively. The siRNA was labeled with a green 5‐carboxyfluorescein (5‐FAM). The nucleus of the cells was labeled with DAPI (blue). The scale bar represents a 20 µm scale.

We constructed self‐assembled TA‐siRNA nanogels for the first time. As shown in **Figure** **1**a, neither the infrared irradiation of the siRNA solution nor the infrared irradiation of the TA solution produces the Tyndall effect, which is due to the uniform dispersion and small size (≤ 1 nm) of the molecules in the solutions. In contrast, TA‐siRNA nanogels clearly show the Tyndall effect, which indicates that the nanoparticles can scatter light. Figure 1b shows that the size of TA‐siRNA nanogels is 50–100 nm. TEM and SEM images show that the nanogels have an irregular sphere‐like morphology (Figure 1b). The chemical structure of TA is characterized by the presence of ester bonds and numerous catechol/phthalate groups. The terminal subunits of TA consist of five catechols and five gallos, both of which can interact with the phosphate backbone of siRNA via hydrogen bonding (Scheme 1). According to a previous study,^[^ ^13^ ^]^ the phenolic groups of TA form reversible hydrogen bonds with the phosphate backbone of DNA to form DNA‐loaded TNA hydrogels. As siRNA and DNA are structurally similar (both are double‐stranded and contain phosphate groups), they form gels by the same mechanism, except siRNA self‐assembles to form nanosized gels because it has a smaller size (20 base pairs) than does DNA (20 000 base pairs). We have verified the cross‐linking mechanism of TA‐siRNA by the FTIR spectra and the 1H NMR spectra as well as the study of temperature‐dependent transition of reversible hydrogen bonding, demonstrating that the main cross‐linking mechanism of TA‐siRNA nanogels is the generation of reversible hydrogen bonds (Figuer S1, Supporting Information). This is consistent with the results of previous studies which investigate the interaction of DNA‐TA.^[^ ^13^ ^]^ Figure 1c shows that the average size of TA‐siRNA nanogels is 84.76 ± 12.35 nm, polydispersity index (PDI) of the nanogels is 0.743, and zeta potential of the nanogels is ‐35.563 ± 2.635 mV. Moreover, the gel fraction of the nanogels is 87.13 ± 4.28%, which indicates that most of the siRNA and TA used for the self‐assembling reaction cross‐linked to form the TA‐siRNA nanogels. The internalization of siRNA into target cells is the primary function of a carrier.^[^ ^14^ ^]^ Figure 1d shows that TA‐siRNA nanogels have numerous phenolic groups on their surfaces, which can interact with specific groups on the cell membrane to promote the cellular adhesion and cellular internalization of the nanogels.^[^ ^15^ ^]^ To verify the cellular internalization of the nanogels, we labeled siRNA with 5‐carboxyfluorescein (5‐FAM). Figure 1e shows that there is slight green fluorescence around the cells incubated with siRNA alone, which indicates that the amount siRNA uptaken by the cells is low. Moreover, the figure demonstrates that TA‐siRNA nanogels promote the cellular internalization of siRNA. After 2 h, green fluorescence is distributed around the cells in the TA‐siRNA nanogel group. After 6 h, the green fluorescence enters the cells through the cell membranes of the cells. The cellular uptake rates of siRNA after cell incubation for 2 h, 6 h, and 12 h are 1.50 ± 0.25%, 3.20 ± 0.36%, and 6.40 ± 0.41%, respectively (*n* = 3). On the other hand, the cellular uptake rates of TA‐siRNA nanogels after cell incubation for 2 h, 6 h, and 12 h are 33.70 ± 2.35%, 58.20 ± 3.14%, and 76.30 ± 3.35%, respectively, which indicates that TA‐siRNA nanogels have a higher transfection efficiency than does siRNA. The quantitative analysis of the confocal microscopy images shown in Figure 1e using ImageJ software revealed a similar trend (Figure S2, Supporting Information): TA‐siRNA nanogels can effectively promote the cellular internalization of siRNA compared with siRNA alone.

### 2.2 Gene‐Silencing Efficiency of TA‐siRNA Nanogels

**Figure 2:**
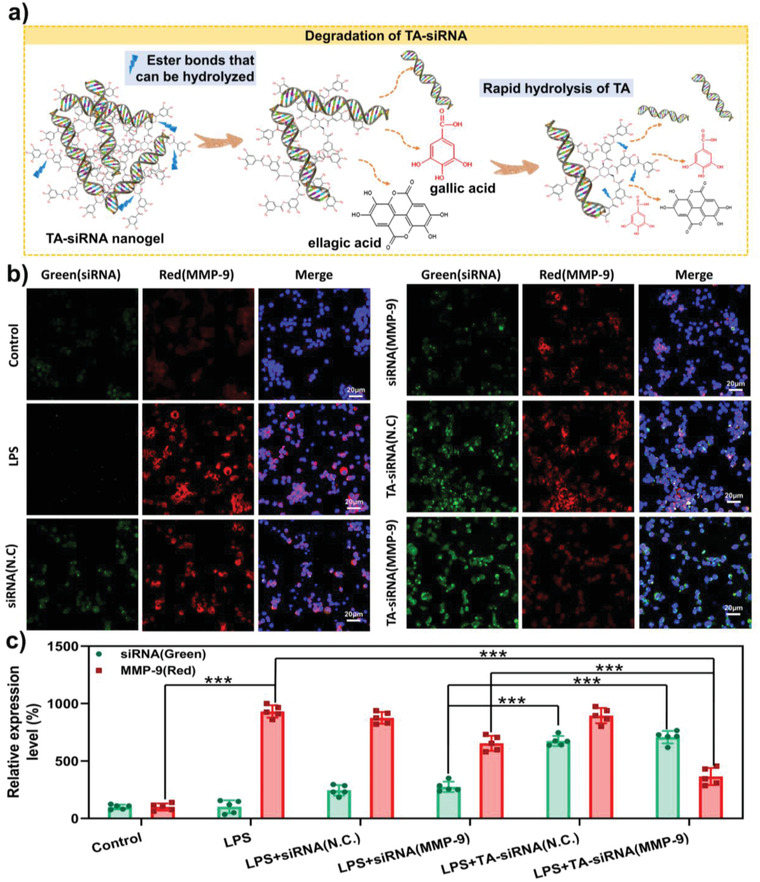
a) Mechanism underlying the release of siRNA from TA‐siRNA nanogels. Through the degradation of TA‐siRNA nanogels, siRNA is gradually released, the essence of which is the hydrolysis of TA (the blue arrow points to the ester bond in the TA structure that can be hydrolyzed). b) The immunofluorescent stain used for assessing the gene‐silencing efficiency of TA‐siRNA nanogels in RAW264.7 cells. The siRNA was labeled with 5‐FAM, and MMP‐9 was labeled with a red immunofluorescent stain. The nucleus of the cells was labeled with DAPI (blue). The scale bar represents a 20 µm scale. c) The quantitative analyses of the levels of siRNA and MMP‐9 in the RAW264.7 cells on the basis of the immunofluorescence staining. The levels of siRNA and MMP‐9 in the cells in the negative control group were set to 100% (***: *p* < 0.001, *n* = 5).

After TA‐siRNA nanogels enter cells, the nanogels are degraded, and biologically‐active monomeric siRNA is released to trigger RNAi‐mediated gene silencing and degradation of target mRNA.^[^ ^16^ ^]^ The hydrolysis of the TA in TA‐DNA hydrogels and release of DNA from the hydrogels was reported by Lee et al.^[^ ^13^ ^]^ As shown in **Figure** **2**a, TA contains several hydrolyzable ester bonds that connect catechol and pyrogallol groups. The hydrolysis of the ester bonds releases catechols and siRNA from TA‐siRNA nanogels. As shown in Figure 2b, lipopolysaccharide(LPS) activates macrophages to increase the level of MMP‐9. The level of red‐fluorescent‐stain‐labeled MMP‐9 in the cells in the LPS‐treated group is high, but MMP‐9 is not expressed in non‐treated cells in the negative control group, which indicates the successful establishment of a model to study the gene‐silencing efficiency of TA‐siRNA nanogels. The siRNA negative control (siRNA (N.C.)) does not silence the *MMP9* gene, while siRNA (MMP‐9) does. Compared to the cellular internalization of siRNA, that of TA‐siRNA nanogels is significantly improved; relatively high intensity of green fluorescence is observed surrounding the cells in the TA‐siRNA (N.C.) nanogel and TA‐siRNA (MMP‐9) nanogel groups. The results are consistent with those presented in Section 2.1. The level of MMP‐9 in the cells in the TA‐siRNA (MMP‐9) nanogel group is substantially lower than those in the LPS, siRNA (N.C.), siRNA (MMP‐9), and TA‐siRNA (N.C.) nanogel groups, indicating that TA‐siRNA (MMP‐9) nanogels have good cellular‐internalization and gene‐silencing efficiencies.

We described a simple and efficient method to prepare non‐toxic self‐assembled TA‐siRNA nanogels whose degradation released siRNA that effectively reduced the LPS‐induced high expression of the *MMP9* gene. The nanogels were loaded into PHTB hydrogels to form PHTB(TA‐siRNA) hydrogels whose siRNA content was 2 optical density (OD) per 20 mL of the hydrogels. The siRNA content of the hydrogels was the effective siRNA concentration obtained on the basis of the cellular‐internalization and gene‐silencing experiments.

We described a simple and efficient method to prepare nontoxic self‐assembled TA‐siRNA nanogels whose degradation released siRNA, which effectively reduced the LPS‐induced high expression of the *MMP9* gene. The nanogels were loaded into PHTB hydrogels to form PHTB(TA‐siRNA) hydrogels whose siRNA content was 2 OD per 20 mL of the hydrogels, which was the effective siRNA concentration obtained on the basis of the cellular‐internalization and gene‐silencing experiments.

### 2.3 Adaptive Properties of PHTB(TA‐siRNA) Hydrogels

The mechanism for the cross‐linkage of PHTB hydrogels has been investigated in detail in the previous study.^[^ ^11^ ^]^ The hydrogels are formed by the cross‐linking of PVA, HLC, TA, and borax through dynamic borate ester bonding and hydrogen bonding. As shown in Scheme 1, the phenolic groups of TA and phosphate groups of siRNA on the surface of TA‐siRNA nanogels can cross‐link with the hydroxyl groups of PVA and hydroxyl groups of TA on the surface of PHTB hydrogels via hydrogen bonding and/or borax‐mediated borate ester bonding. The results of rheological experiments suggested that the cross‐linking of TA‐siRNA nanogels and PHTB hydrogels slightly enhanced the mechanical properties of the resulting PHTB(TA‐siRNA) hydrogels (Figure S3, Supporting Information). The damping coefficient (*G*″/*G*′), which is the ratio of the loss modulus (*G*″) to the storage modulus (*G*′), of PHTB(TA‐siRNA) hydrogels was 0.982 ± 0.034; therefore, the hydrogels have a combination of the elasticity of gel, and the fluidity of a fluid, and good shape adaptability.^[^ ^11^ ^]^ The addition of TA‐siRNA nanogels to PHTB hydrogels slightly decreased the conductivity of PHTB(TA‐siRNA) hydrogels compared to that of PHTB hydrogels (Figure S3, Supporting Information) because the addition of TA‐siRNA nanogels reduces the amount of borax that reacts with PVA or TA to form borate ester bonds and therefore reduces the amount of conductive ions. Moreover, the increase in the cross‐link density of PHTB(TA‐siRNA) hydrogels may hinder the directional movement of charged ions, which decreases the conductivity of the hydrogels.

**Figure 3:**
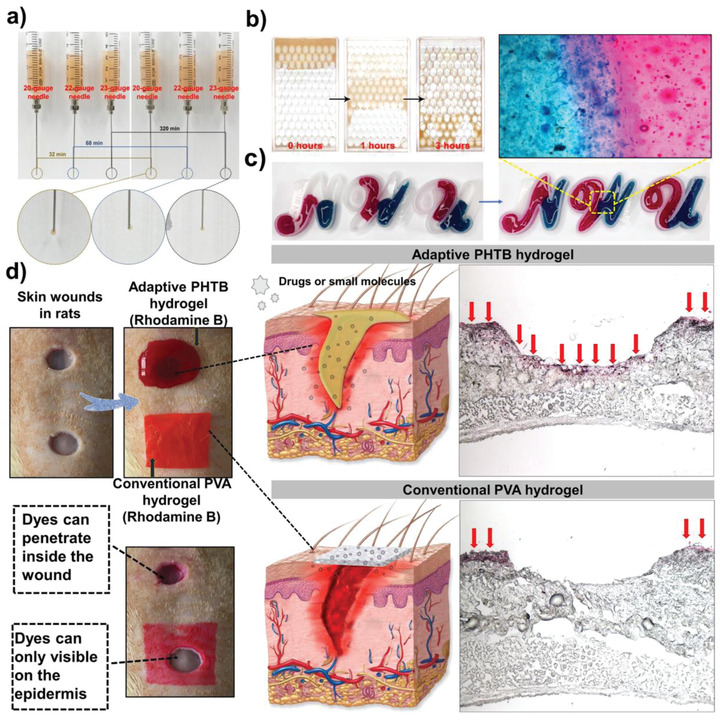
a) The adaptive properties of PHTB(TA‐siRNA) hydrogels indicated by the ability of the hydrogels to escape pinheads. The inner diameters of the 20‐gauge, 22‐gauge, and 23‐gauge needles were 0.60 mm, 0.41 mm, and 0.34 mm, respectively. b) The adaptive properties of the hydrogels indicated by the ability of the hydrogels to escape spherical gaps. c) The adaptive and self‐healing properties of the hydrogels. d) The differences in the release of drugs or genes from the conventional dressing and PHTB(TA‐siRNA) hydrogel dressing.

The macroscopic shape adaptability of PHTB(TA‐siRNA) hydrogels is shown in **Figure** **3**a. The hydrogels leaked out of 20‐gauge, 22‐gauge, and 23‐gauge needles on minutes 32, 68, and 320, respectively, which indicates the good fluidity of the hydrogels. As shown in Figure 3b, when PHTB(TA‐siRNA) hydrogels are placed in a glass container filled with spheres, the hydrogels immediately fill the upper layers of the spheres and gradually move downward due to gravity. The hydrogels reach the bottom layers of the spheres after 3 h. When PHTB(TA‐siRNA) hydrogels had been stained with rhodamine B (RhB) and alcian blue before being squeezed in separate molds (Figure 3c), the hydrogels slowly filled the molds under no external forces and exhibited self‐healing properties at the interface between the molds. The adaptive and self‐healing properties of PHTB(TA‐siRNA) hydrogels are contributed by the dynamic borate ester bonds and hydrogen bonds in the hydrogels, which can quickly restore the network structure of the hydrogels owing to the inherent dynamic equilibria of the bonds.

Adaptive hydrogels can adapt to shapes of wounds and fill deep wounds. This could suggest that drug‐loaded adaptive hydrogels can release drugs in deep wounds. RhB, which was selected as a model drug, was loaded into adaptive PHTB hydrogels and conventional PVA hydrogels with a fixed shape. Circular wounds that were 2 cm apart were established in the backs of rats, and the RhB‐loaded conventional PVA hydrogels and RhB‐loaded PHTB hydrogels were applied to the wounds. The hydrogel‐based dressings were removed after 30 min. As shown in Figure 3d, red color, which corresponds to the color of RhB, is observed on the epidermis of the wounds to which the PVA hydrogels were applied, but red color is not observed inside the wounds. On the other hand, red color is observed inside the wounds to which the PHTB hydrogels were applied. This indicates that the PHTB hydrogels are able to fill the wounds and release RhB inside the wounds.

### 2.4 Responsiveness of PHTB(TA‐siRNA) Hydrogels to ROS Levels and the Release of Genes from the Hydrogels

Cellular respiration and metabolic processes are major sources of ROS.^[^ ^17^ ^]^ In response to hyperglycemia, which results from an abnormal glucolipid metabolism, macrophages and neutrophils produce ROS that causes oxidative stress in diabetic chronic wounds.^[^ ^18^ ^]^ Under normal conditions, the level of ROS in the human body is low; the concentration of H_2_O_2_ in blood plasma ranges from 1 × 10^−6^ to 8 × 10^−6^ m (average: 3 × 10^−6^ m),^[^ ^19^ ^]^ and the average concentration of ROS in healthy cells is 1 × 10^−6^ m. In comparison, the concentration of ROS in activated macrophages ranges from 10 × 10^−6^ to 1000 × 10^−6^ m.^[^ ^20^ ^]^ An excess amount of ROS induces a strong inflammatory response and inhibits macrophage functions, blood‐vessel formation, and cell regeneration, therefore, prevents the repair of wounds.^[^ ^4^ ^]^ ROS‐responsive drug‐delivery systems are promising carriers of therapeutic substances because high levels of ROS in oxidative physiological microenvironments may trigger drug release.^[^ ^20^ ^]^ Under physiological pH and temperature, H_2_O_2_ can oxidize boronate esters.^[^ ^21^ ^]^ To investigate the efficacy of PHTB(TA‐siRNA) hydrogels to repair diabetic chronic wounds, in which the degree of oxidative stress is high, we examined the disintegration of the hydrogels and release of siRNA from the hydrogels due to different H_2_O_2_ concentrations (10–1000 × 10^−6^ m).

**Figure 4:**
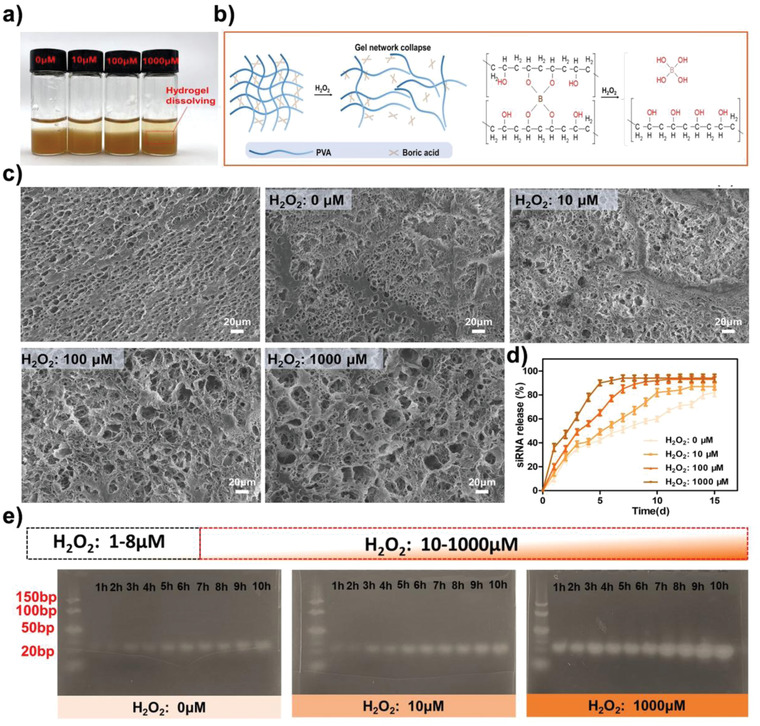
a) The addition of different concentrations of hydrogen peroxide (H_2_O_2_) to PHTB(TA‐siRNA) hydrogels and b) mechanism for the response of the hydrogels to ROS. c) The SEM images of the freeze‐dried hydrogels after the addition of the different concentrations of H_2_O_2_. d) The rates of the release of siRNA from the hydrogels after the addition of the different concentrations of H_2_O_2_ to the hydrogels (*n* = 3). e) The agarose gel electrophoresis analysis of the siRNA released from the hydrogels after the addition of the different concentrations of H_2_O_2_.

As shown in **Figure** **4**a, in the absence of H_2_O_2_, the liquid in the sample vial remains transparent. After the reactions between PHTB(TA‐siRNA) hydrogels and different concentrations of H_2_O_2_ (10 × 10^−6^, 100 × 10^−6^, and 1000 × 10^−6^ m) for 30 min, the liquids in the sample vials show different intensities of yellow color (Figure 4b), which correspond to the different degrees of the oxidation of the borate ester bonds in the hydrogels and release of TA from the hydrogels. In the sample vial to which the 1000 × 10^−6^ m H_2_O_2_ solution was added, the interface between the liquid and the hydrogels blurs, which indicates that the H_2_O_2_ concentration enhances the oxidation of borate ester bonds and dissolution of the hydrogels. The microscopic morphology of the freeze‐dried PHTB(TA‐siRNA) hydrogels after the addition of different concentrations of H_2_O_2_ (Figure 4c) indicated that the higher the H_2_O_2_ concentration, the larger the pore size of the hydrogels. This indicates that borate ester bonds in the hydrogels are degraded in the oxidative environment, which decreases the cross‐link density and increases the pore size of the hydrogels. Moreover, the higher the H_2_O_2_ concentration, the larger the range of the pore size distribution of the hydrogels (Figure S4, Supporting Information), which indicates that the network structure of the hydrogels gradually disintegrates in the oxidative environment.

To verify the influence of the proinflammatory concentrations of H_2_O_2_ (10–1000 × 10^−6^ m) on the release of siRNA from PHTB(TA‐siRNA) hydrogels, we used agarose gel electrophoresis to examine the effects of the H_2_O_2_ concentrations on the amount of siRNA released from the hydrogels. In the absence of H_2_O_2_, a small amount of siRNA was released from the hydrogels over time (Figure 4e) due to the hydrolysis of the borate ester bonds in the hydrogels. The hydrolysis of the bonds releases TA‐siRNA nanogels, and the hydrolysis of the ester bonds that link the catechol and pyrogallol in the nanogels releases siRNA. Under the high concentrations of H_2_O_2_, borate ester bonds in PHTB(TA‐siRNA) hydrogels are rapidly oxidized, which disintegrates the hydrogels and releases a large amount of TA‐siRNA nanogels and a large amount of siRNA. Therefore, the higher the H_2_O_2_ concentration, the larger the bands in the agarose gel (Figure 4e) due to the higher amount of siRNA released from PHTB(TA‐siRNA) hydrogels. As shown in Figure 4d, at the proinflammatory concentration range of H_2_O_2_, the higher the H_2_O_2_ concentration, the higher the release rate of siRNA. Therefore, in response to high ROS levels in chronic wounds, PHTB(TA‐siRNA) hydrogels may release a higher amount of siRNA to reduce the high production of MMP‐9 in the wounds.

### 2.5 Efficacy of PHTB(TA‐siRNA) Hydrogels to Scavenge ROS

**Figure 5:**
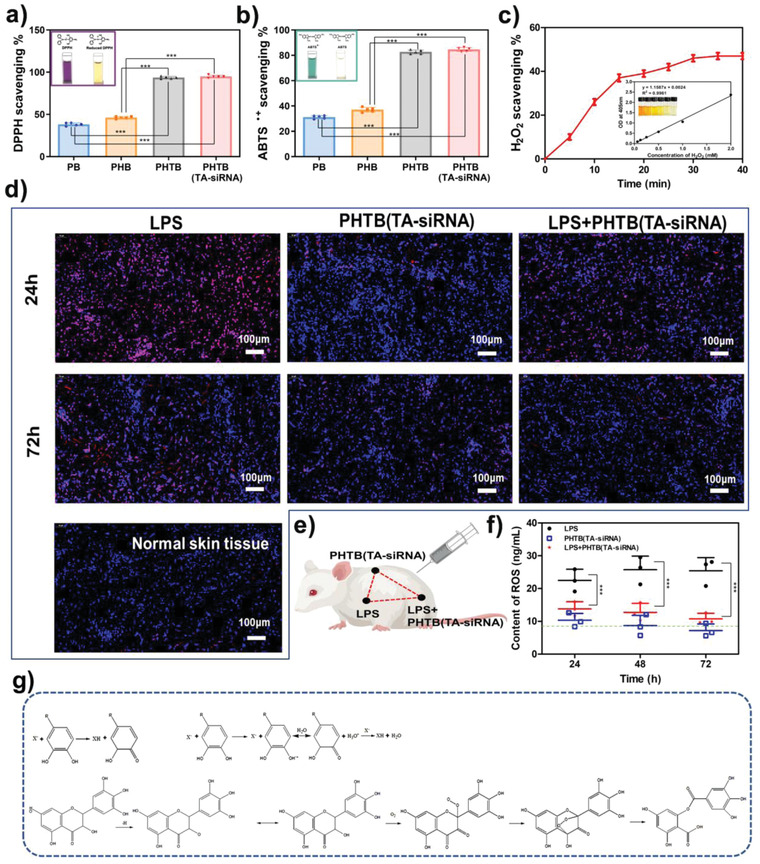
The scavenging effects of PHTB(TA‐siRNA) hydrogels on a) the DPPH radical, b) ABTS^∙+^ radical, and c) H_2_O_2_ radical (***: *p* < 0.001, *n* = 5). d) The levels of subcutaneous ROS in rats treated with PHTB(TA‐siRNA) hydrogels. The subcutaneous‐ROS levels were detected using a dihydroethidium (DHE) fluorescent probe. The violet color indicates the content of intracellular ROS, and the blue color represents 4',6‐diamidino‐2‐phenylindole (DAPI)‐stained cell nucleus. The scale bar represents a 100 µm scale. e) Schematic diagram of the in vivo experiments to detect the scavenging of ROS by PHTB(TA‐siRNA) hydrogels. f) The levels of ROS in tissues that received different treatments (***: *p* < 0.001, *n* = 3). The green dashed line indicates the amount of ROS in normal skin tissues. g) The mechanism for the antioxidant properties of PHTB(TA‐siRNA) hydrogels.

Antioxidant properties of PHTB(TA‐siRNA) hydrogels were initially investigated in vitro using the DPPH radical scavenging assay and ABTS^∙+^ radical scavenging assay. As shown in **Figure** **5**a,b, the radical scavenging effects of PHTB hydrogels and PHTB(TA‐siRNA) hydrogels are superior to those of PB hydrogels and PHB hydrogels. The radical scavenging rates of PHTB hydrogels and PHTB(TA‐siRNA) hydrogels on the DPPH radical and ABTS^∙+^ radical are higher than 80%. Figure 5c shows the radical scavenging rate of PHTB(TA‐siRNA) hydrogels on the H_2_O_2_ radical, which reaches equilibrium (46.56 ± 3.21%) after 30 min. These results indicate the excellent antioxidant properties of PHTB(TA‐siRNA) hydrogels.

We further explored the ROS scavenging effect of PHTB(TA‐siRNA) hydrogels in vivo in microenvironments whose levels of ROS were high. LPS is commonly employed to induce inflammation and ROS production.^[^ ^22^ ^]^ The quantification of the level of subcutaneous ROS in skin tissues using an ROS fluorescent probe (Figure 5d) showed that normal skin tissues and the skin tissues injected with PHTB(TA‐siRNA) hydrogels had negligible amounts of ROS, which indicates that the injection of the hydrogels does not induce the production of ROS in normal skin tissues. The injection of PHTB(TA‐siRNA) hydrogels into LPS‐induced skin tissues significantly reduced the amounts of ROS in the skin tissues, which indicates the ROS scavenging effect of the hydrogels. The quantification of the levels of subcutaneous ROS in skin tissues using an ROS assay kit (Figure 5f) revealed similar results to those obtained by using the ROS fluorescent probe. The ROS levels in the normal skin tissues were lower than 10 ng mL^−1^, while those in the LPS‐induced skin tissues were two times as high as those in the normal skin tissues. In contrast, PHTB(TA‐siRNA) hydrogels effectively reduced the levels of ROS in the LPS‐induced skin tissues. The results of the in vitro and in vivo experiments to detect the ROS scavenging effect of PHTB(TA‐siRNA) hydrogels suggested that the hydrogels effectively reduced the high levels of ROS in chronic wounds.

The excellent antioxidant properties of PHTB(TA‐siRNA) hydrogels are attributed to the excellent antioxidant properties of TA. TA is a plant polyphenol that is a known ROS scavenger.^[^ ^23^ ^]^ ROS scavenging effects of polyphenols are mainly attributed to the phenolic groups in the polyphenols (Figure 5g). Polyphenols can scavenge ROS by providing hydrogen atoms, which quench ROS or transfer electrons to them, or transferring electrons to ROS.^[^ ^24^ ^]^ Thus, due to their TA content, PHTB(TA‐siRNA) hydrogels exhibit good in vitro and in vivo antioxidant properties, which may be beneficial to the repair of chronic wounds by using the hydrogels.

### 2.6 The Effect of ES on the Release of TA‐siRNA Nanogels and Cellular Internalization

**Figure 6:**
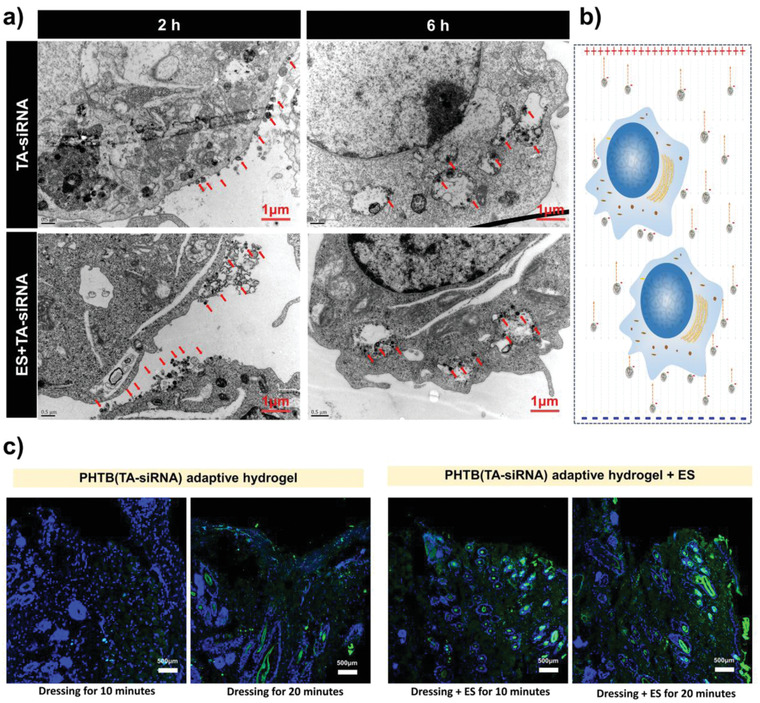
a) The TEM images of the adhesion of TA‐siRNA nanogels to macrophages and cellular uptake of the nanogels. b) The effect of an electric field on the adhesion and cellular uptake of the nanogels. c) Effect of ES therapy on the release of siRNA from PHTB(TA‐siRNA) hydrogels during wound application. The siRNA was labeled with a green immunofluorescent stain (i.e., 5‐FAM), and the blue color represents DAPI‐stained nucleus. The scale bar represents a 500 µm scale.

In our previous study, we showed that PHTB hydrogels act as conductive media that improve the efficacy of ES therapy and promote intercellular signaling, cellular migration, and vascular regeneration.^[^ ^11^ ^]^ As ES therapy generates an electric field between tissues and influences the movement of charged particles, we hypothesized that the therapy promotes the release of TA‐siRNA nanogels from PHTB(TA‐siRNA) hydrogels and cellular uptake of the nanogels. The red arrow shown in **Figure** **6**a indicates that in the absence of an electric field, TA‐siRNA nanogels interact with proteins on the cell membrane. The endocytic vesicles produced by the cell membrane encapsulate the nanogels and transport the nanogels through the cell membrane. Under the action of an electric field, the number of TA‐siRNA nanogels aggregated on the cell membrane increases, and the cellular uptake rate of the nanogels increases from 35.10 ± 1.12% to 75.40 ± 2.36% in 2 hours (*n* = 3), which indicates that an electric field promotes the cellular endocytosis of the nanogels.

We further investigated whether ES therapy enhanced the wound‐repair efficacy of PHTB(TA‐siRNA) hydrogels. As shown in Figure 6c, we labeled siRNA with 5‐carboxyfluorescein and then prepared PHTB(TA‐siRNA) hydrogels and applied it to wounds. There is no green‐immunofluorescent‐stain‐labeled siRNA in the wound to which PHTB(TA‐siRNA) hydrogels were applied for 10 min, and after the application of the hydrogels for 20 min, siRNA is observed in the skin tissues of the wound. Applying PHTB(TA‐siRNA) hydrogels in combination with ES therapy to a wound produces stronger green fluorescence than does applying the hydrogels alone to a wound for a same time. The amount of siRNA released from the hydrogels increases after the application of the combination therapy for 20 min, which indicates that ES therapy promotes the release of siRNA from PHTB(TA‐siRNA) hydrogels to repair the wound. Chen et al. investigated the effect of ES on the release of human bone morphogenetic protein gene 4 (hBMP‐4) from electroactive tissue‐engineered scaffolds. It was hypothesized that the current stimulated the movement of the charged gene carrier composites, thereby accelerating the release of hBMP‐4.^[^ ^25^ ^]^ Kang et al. immobilized PVA‐heparin on a highly electrical conductive PPY polymer, and the release behavior of heparin from PPY‐PVA‐heparin under constant electrical current was studied.^[^ ^26^ ^]^ This study explained the mechanism by which ES promoted the release of heparin by altering the interaction between the polyanionic heparin molecule and the positively charged PPY backbone in the presence of an electric field, thereby influencing the movement of the heparin molecule and controlling the release. Previous studies have demonstrated that ES therapy can promote blood circulation.^[^ ^27^ ^]^ Therefore, we hypothesized that ES could affect the movement of charged nanogels to promote the release of charged nanogels from the hydrogel and cellular uptake rate. Besides, the ES therapy could enhance the circulation of blood and flow of tissue fluids, which further promotes the degradation of PHTB(TA‐siRNA) hydrogels and TA‐siRNA nanogels, facilitating the release of siRNA. Therefore, ES enhances the release of siRNA from PHTB(TA‐siRNA) hydrogels and cellular uptake of the siRNA.

### 2.7 Treatments of Chronic Wounds in Diabetic Rats with the Combination of PHTB(TA‐siRNA) Hydrogels and ES Therapy

**Figure 7:**
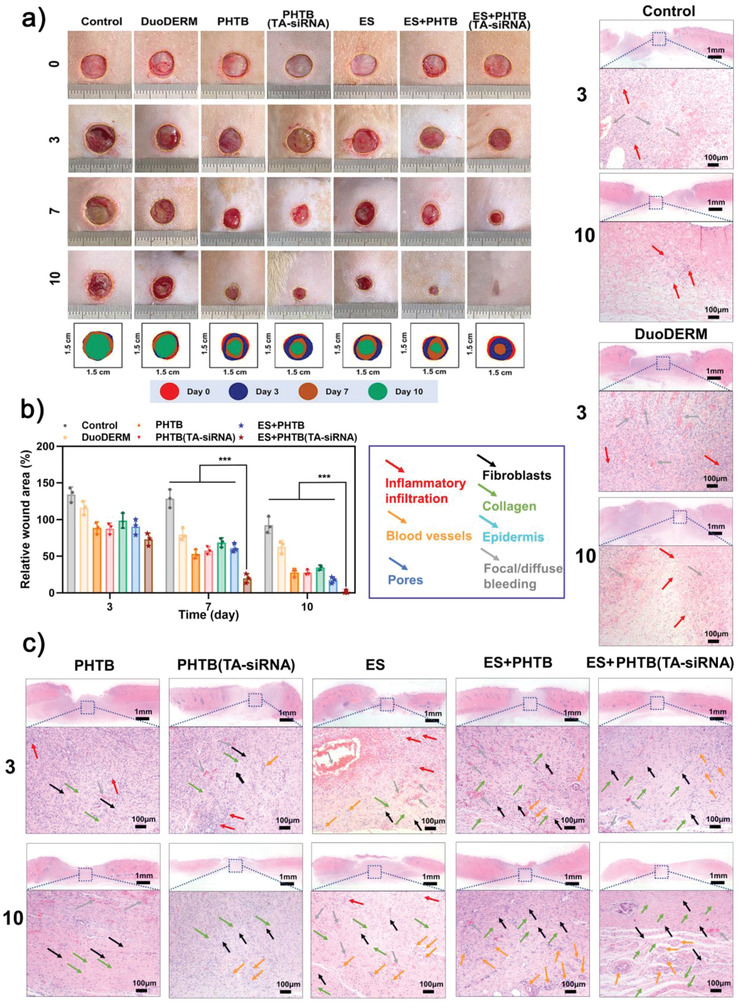
a) Full‐thickness skin defects in diabetic rats and the healing trajectory of the defects. b) The healing rates of the defects on days 3, 7, and 10 (***: *p* < 0.001, *n* = 3). c) The low‐magnification (scale bar: 1 mm) and high‐magnification (scale bar: 100 µm) of H&E staining images of the defects on days 3 and 10.

PHTB(TA‐siRNA) hydrogels responded to high ROS levels in chronic wounds and released TA, which scavenged ROS, and TA‐siRNA nanogels, which released siRNA to silence the *MMP9* gene that is highly expressed in the proinflammatory microenvironments of chronic wounds. The hydrogels served as conductive media that improved the transmission of the electric current and thus improved the efficacy of ES therapy. On the other hand, ES therapy promoted the release of TA‐siRNA nanogels from PHTB(TA‐siRNA) hydrogels and the release of siRNA from the nanogels. Moreover, the therapy promoted the cellular internalization of the nanogels and siRNA. The effects of ES therapy and PHTB (TA‐siRNA) hydrogel on wounds are not independent, but interact with each other. **Figure** **7**a shows the ability of the combination of ES therapy and PHTB(TA‐siRNA) hydrogels to promote the repair of full‐thickness skin defects (diameter: 8 mm) in the backs of diabetic rats. On days 3 and 7, the defects in the diabetic rats in the control group show a significant number of white abscesses, which indicates the severity of inflammation in the defects.^[^ ^28^ ^]^ On day 7, the areas of the defects in the diabetic rats in the PHTB hydrogel group, PHTB(TA‐siRNA) hydrogel group, ES therapy‐PHTB hydrogel group, and ES therapy‐PHTB(TA‐siRNA) hydrogel group are smaller than those in the diabetic rats in the control group, ES therapy group, and commercial dressing group; the defects in the diabetic rats in the ES therapy‐PHTB(TA‐siRNA) hydrogel group have the smallest area, which indicates the highest efficacy of the combination therapy to promote wound healing. Figure 7b shows that on day 3, the areas of the defects in the diabetic rats in the control group, commercial dressing group, and ES therapy group are greater than the initial areas of the defects, which is due to infection‐induced or hyperglycemia‐induced inflammation that results in abscesses on the defects. The defects in the diabetic rats in the ES therapy–PHTB(TA‐siRNA) hydrogel group are completely healed on day 10. On the other hand, the defects in the diabetic rats in the control group are completely healed on day 19. Thus, the combination of ES therapy and PHTB(TA‐siRNA) hydrogels reduces the repair time of full‐thickness skin defects in diabetic rats by 47.4%. These results indicate that the commercial dressing and ES therapy cannot effectively repair complex and severe diabetic wounds. The combination of ES therapy and PHTB(TA‐siRNA) hydrogels promotes the healing of full‐thickness skin defects in diabetic rats more effectively than do PHTB hydrogels and PHTB(TA‐siRNA) hydrogels. Although PHTB(TA‐siRNA) hydrogels may effectively remove MMP‐9 from the defects, the combination therapy may promote cell migration, intercellular signaling, blood circulation, and vascular regeneration. Moreover, ES therapy may promote not only the release of TA‐siRNA nanogels from PHTB(TA‐siRNA) hydrogels, but also the release siRNA from the nanogels and cellular internalization of the nanogels and siRNA. Therefore, the combination therapy had the most effective therapeutic effect among wound‐healing agents investigated in this work.

H&E staining allows researchers to observe the skin structure, which consists of capillary‐rich fibrous connective tissues, inflammatory infiltration, fibroblasts, and collagen fibers.^[^ ^29^ ^]^ On day 3, a large number of inflammatory cells were detected in the defects in the diabetic rats in the control group, commercial dressing group, and ES therapy group (Figure 7c and Figure S5, Supporting Information). On the other hand, there were fewer inflammatory cells in the defects in the diabetic rats in the PHTB hydrogel group, PHTB(TA‐siRNA) hydrogel group, ES therapy‐PHTB hydrogel group, and ES therapy‐PHTB(TA‐siRNA) hydrogel group. These results indicate that both PHTB hydrogels and PHTB(TA‐siRNA) hydrogels effectively reduce inflammation in full‐thickness skin defects in diabetic rats. A large number of small blood vessels were formed in the dermal tissues of the defects in the diabetic rats in the ES therapy group, ES therapy‐PHTB hydrogel group, and ES therapy‐PHTB(TA‐siRNA) hydrogel group, which indicates that ES therapy promotes angiogenesis. On day 10, the combination of ES therapy and PHTB hydrogels and that of ES therapy and PHTB(TA‐siRNA) hydrogels fully recovered the epidermal structures and blood vessels in the defects in the diabetic rats in the ES therapy‐PHTB hydrogel group and ES therapy‐PHTB(TA‐siRNA) hydrogel group. Moreover, other attachments were observed in the dermis of the defects. These results indicate the effectiveness of the combinations of ES therapy and the hydrogels to repair full‐thickness skin defects in diabetic rats.

The efficacy of the combination of ES therapy and PHTB(TA‐siRNA) hydrogels to repair burns in the diabetic rats was investigated, and the results were shown in the supporting information. The burns in the diabetic rats in the ES therapy‐PHTB(TA‐siRNA) hydrogel group were completely closed on day 20, while the burns in the diabetic rats in the control group were completely closed on day 33 (Figures S6 and S7, Supporting Information). Therefore, the combination therapy reduced the repair time of burns in diabetic rats by 39.4%, which indicated the high efficacy of the combination therapy to repair burns in diabetic rats.

### 2.8 Mechanisms for Anti‐Inflammatory, Antioxidant, and Promacrophage Polarization Properties of the Combination of ES Therapy and PHTB(TA‐siRNA) Hydrogels

**Figure 8:**
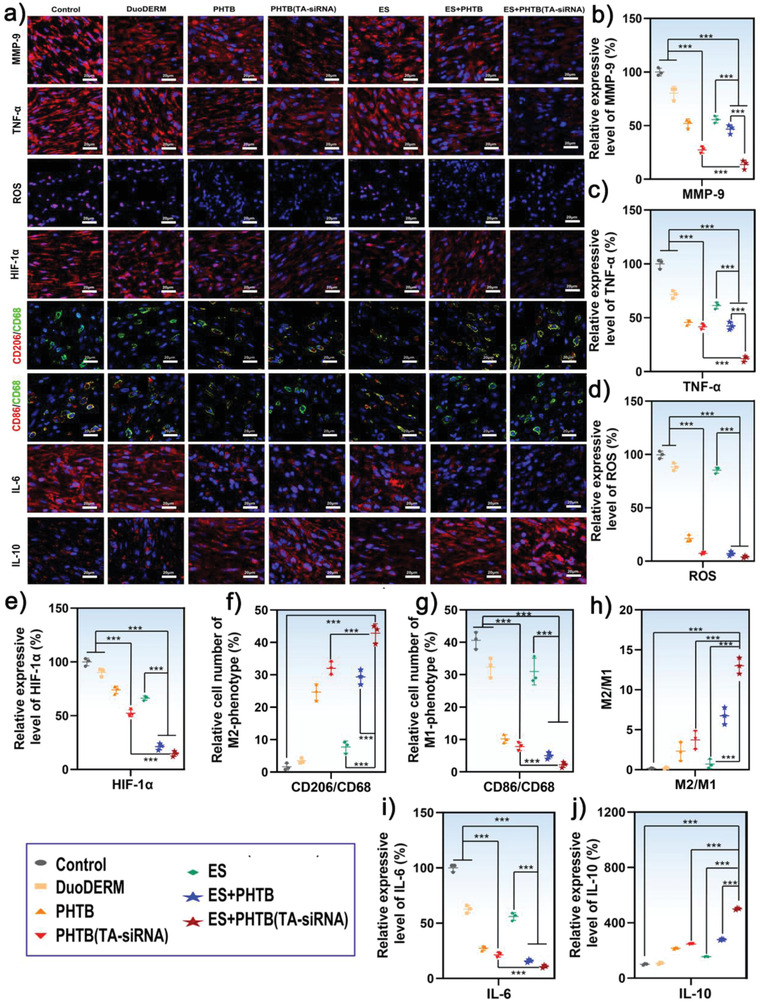
a) The immunofluorescent staining of the MMP‐9, TNF‐*α*, HIF‐1*α*, IL‐10, and IL‐6 expressed in diabetic chronic wounds and the labeling of the ROS and macrophage phenotypic markers produced in the skin tissues. The blue color represents DAPI‐stained cell nucleus. The ROS was labeled with a DHE fluorescent probe (violet color). Immunofluorescence double staining of CD68‐labeled (green) macrophages, and CD206‐labeled (red) M2‐type macrophages. Immunofluorescence double staining of CD68‐labeled (green) macrophages, and CD86‐labeled (red) M1‐type macrophages. The analyses were performed on day 7, and the scale bar represents a 20 µm scale. The quantitative analyses for the intensities of the fluorescent signals produced by b) MMP‐9, c) TNF‐*α*, d) ROS, e) HIF‐1*α*. The intensities of the fluorescent signals in diabetic chronic wounds in the control groups were set to 100% (**: *p* < 0.01, ***: *p* < 0.001, *n* = 3).f) The population of double‐positive M1 macrophages and g) that of double‐positive M2 macrophages (***: *p* < 0.001, *n* = 3). h) The population ratios of M2 macrophages to M1 macrophages (***: *p* < 0.001, *n* = 3). The quantitative analyses for the intensities of the fluorescent signals produced by i) IL‐6, and j) IL‐10. (***: *p* < 0.001, *n* = 3).

We further analyzed the roles of the anti‐inflammatory and antioxidant properties of PHTB(TA‐siRNA) hydrogels in the wound healing properties of the combination of ES therapy and the hydrogels. The overproduction of MMP‐9 impairs the formation of granulation tissues and hinders the closure of wounds.^[^ ^30^ ^]^ TA‐siRNA nanogels carry siRNA that may reduce the expression of the *MMP9* gene. Therefore, we used immunofluorescent staining to investigate the efficacy of PHTB(TA‐siRNA) hydrogels to silence the expression of the *MMP9* gene in diabetic chronic wounds As shown in **Figure** **8**a,b, the MMP‐9 is highly expressed in the skin defects of the diabetic rats in the control group, and the expression of the gene significantly decreases in the full‐thickness skin defects of the diabetic rats in the PHTB(TA‐siRNA) hydrogel group and ES therapy‐PHTB(TA‐siRNA) hydrogel group. These results indicate that the siRNA in TA‐siRNA nanogels effectively interferes with the mRNA of the *MMP9* gene, and ES therapy promotes the release and cellular internalization of TA‐siRNA nanogels (Section 2.6). TNF‐*α* is a common proinflammatory factor whose amount corresponds to the level of inflammation in tissues. The high amounts of TNF‐*α* in diabetic chronic wounds in the control groups and commercial dressing groups indicate that the commercial dressing cannot inhibit inflammation in the diabetic chronic wounds (Figure 8c). On the other hand, PHTB hydrogels and PHTB(TA‐siRNA) hydrogels and the combinations of ES therapy and the hydrogels reduce inflammation in diabetic chronic wounds. The anti‐inflammatory effects of the hydrogels are attributed to the TA and HLC. The amounts of TNF‐*α* in the diabetic chronic wounds in the ES therapy‐PHTB(TA‐siRNA) hydrogel groups are the lowest among the amounts of TNF‐*α* in the diabetic chronic wounds in the different groups (Figure 8c). Although PHTB hydrogels and PHTB(TA‐siRNA) hydrogels contain the same anti‐inflammatory components (TA and HLC), the TA‐siRNA nanogels in PHTB(TA‐siRNA) hydrogels can reduce the production of MMP‐9 and degradation of cellular proteins by MMP‐9. Thus, the nanogels can improve the anti‐inflammatory effect of PHTB(TA‐siRNA) hydrogels.

An excess amount of ROS induces a strong inflammatory response and inhibits macrophage functions, blood‐vessel formation, and cell regeneration. Therefore, it prevents the healing of wounds.^[^ ^4^ ^]^ Through in vitro and in vivo experiments, we demonstrated that PHTB(TA‐siRNA) hydrogels had an excellent ROS‐scavenging efficacy. Therefore, we examined the ROS levels in the diabetic chronic wounds. The results are shown in Figure 8a,d. The ROS levels in the diabetic chronic wounds in the PHTB hydrogel groups, PHTB(TA‐siRNA) hydrogel groups, ES therapy‐PHTB hydrogel groups, and ES therapy‐PHTB(TA‐siRNA) hydrogel groups are significantly lower than those in the diabetic chronic wounds in the control groups. The results indicate that the TA in PHTB(TA‐siRNA) hydrogels effectively removes ROS. Hypoxic conditions due to high levels of ROS induce the high expression of hypoxia‐inducible factor 1 (HIF‐1).^[^ ^31^ ^]^ HIF‐1 consists of two subunits: HIF‐1*α* and HIF‐1*β*. Under conditions in which cellular oxygen levels are normal, HIF‐1*α* is almost undetectable. However, under hypoxic conditions, the level of HIF‐1*α* in cells increases rapidly. Hypoxic conditions inhibit the rapid ubiquitination and degradation of HIF‐1*α* by the proteasome, which increases levels of HIF‐1*α* in cells. Thus, HIF‐1*α* is an indicator of hypoxic conditions in cells.^[^ ^32^ ^]^ As shown in Figure 8a,e, on day 7, the diabetic chronic wounds in the control group have the highest amount of HIF‐1*α* among the diabetic chronic wounds in the different groups, which indicates that the wounds in the control group are in a hypoxic state. In contrast, the wounds in the ES therapy–PHTB(TA‐siRNA) hydrogel group have the lowest amount of HIF‐1*α*, which indicates that the combination of ES therapy and PHTB(TA‐siRNA) hydrogels alleviate the hypoxic microenvironments of the wounds. The reduction in the degree of hypoxia in the diabetic chronic wounds may be attributed to the ROS‐scavenging effect of TA and enhanced oxygen transportation through new blood vessels whose formation (H&E staining) is promoted by ES therapy.

Macrophages are the key regulators of the immune response and wound‐healing process. To investigate the mechanism by which the combination of ES therapy and PHTB(TA‐siRNA) hydrogels modulated macrophage polarization and alleviated inflammation, we used immunofluorescent staining to delineate macrophage phenotypes in tissues. Typically, macrophages can be activated in response to the microenvironment and can be converted to different polarization states: classically activated macrophages (the M1 phenotype) or alternatively activated macrophages (the M2 phenotype).^[^ ^33^ ^]^ M2 macrophages release pro‐angiogenic factors and anti‐inflammatory cytokines. Therefore, they are important to the vascularization of wounds.^[^ ^34^ ^]^ However, diabetes impairs the functions of macrophages and inhibits the phenotypic switch from pro‐inflammatory M1 macrophages to anti‐inflammatory M2 macrophages. CD68 is the most reliable marker of macrophages. We labeled macrophages with CD68, and M1 macrophages with CD86, and M2 macrophages with CD206. Figure 8a shows immunofluorescent staining of the CD86(red)/CD68(green)‐labeled M1 macrophages and CD206(red)/CD68(green)‐labeled M2 macrophages. Many M1 macrophages and almost no M2 macrophages were detected in the diabetic chronic wounds in the control group, commercial dressing group, and ES therapy group (Figure 8a,f,g). On the other hand, M2 macrophages were detected in the diabetic chronic wounds in the PHTB hydrogel group, PHTB(TA‐siRNA) hydrogel group, ES therapy‐PHTB hydrogel group, and ES therapy‐PHTB(TA‐siRNA) group. The wounds in the ES therapy‐PHTB(TA‐siRNA) hydrogel group have the highest number of M2 macrophages among the wounds in the different groups. The population ratios of M2 macrophages to M1 macrophages in the diabetic chronic wounds in the ES therapy‐PHTB(TA‐siRNA) hydrogel groups were significantly higher than those in the diabetic chronic wounds in the other groups (Figure 8h). These results indicate that the combination of ES therapy and PHTB(TA‐siRNA) hydrogels can modulate macrophage polarization due to the excellent anti‐inflammatory and antioxidant effects of TA and HLC^[^ ^15^, ^35^ ^]^ and *Mmp9*‐gene silencing effect of TA‐siRNA nanogels. In addition, an electric field can inhibit Akt2‐IRF5 signaling and create a favorable osteo‐immunomodulatory environment that reduces the secretion of IL‐6 and induces the polarization of M1 macrophages to M2 macrophages under hyperglycemic conditions.^[^ ^36^ ^]^ To further investigate the effect of the combination of ES therapy and PHTB(TA‐siRNA) hydrogels on macrophage polarization, we used immunofluorescent staining to detect the amount of IL‐6, which is a specific proinflammatory factor expressed by M1 macrophages, and that of IL‐10, which is a specific anti‐inflammatory factor expressed by M2 macrophages. The results are shown in Figure 8a,i,j. High amounts of IL‐6 and low amounts of IL‐10 were detected in the diabetic chronic wounds in the control group, commercial dressing group, and ES therapy group. Different amounts of IL‐10 were detected in the diabetic chronic wounds in the PHTB hydrogel group, PHTB(TA‐siRNA) hydrogel group, ES therapy‐PHTB hydrogel group, and ES therapy‐PHTB(TA‐siRNA) hydrogel group; the amounts of IL‐10 in the wounds in the diabetic rats in the ES therapy‐PHTB(TA‐siRNA) hydrogel group were higher than those in the wounds in the diabetic rats in the other groups, which indicates that the combination therapy not only promotes the polarization of macrophages, but also shifts the state of the tissue microenvironment from a pro‐inflammatory state to an anti‐inflammatory state. Thus, the combination therapy reduces inflammation and promotes wound healing. ES therapy‐PHTB(TA‐siRNA) hydrogel also showed the best therapeutic effect in the burns of the diabetic rats, with good anti‐inflammatory, antioxidant, and pro‐macrophage polarization properties (Figure S8, Supporting Information)

### 2.9 Mechanisms for Procollagen Production and Provascularization Properties of the Combination of ES Therapy and PHTB(TA‐siRNA) Hydrogels

**Figure 9:**
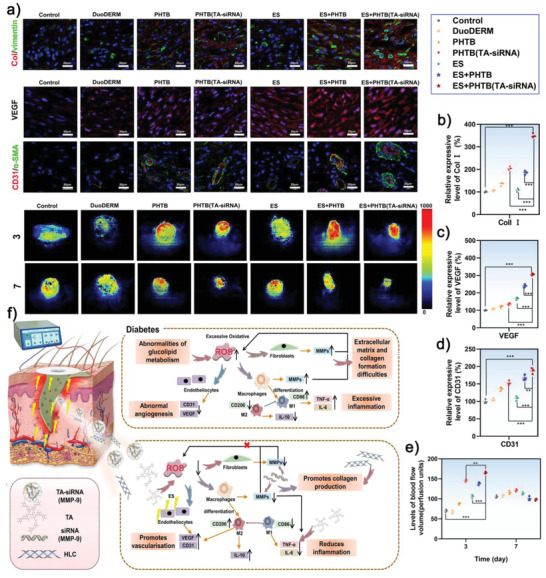
a) The immunofluorescent staining of the Col I, VEGF, and CD31 expressed on day 7 in the diabetic chronic wounds and the laser speckle contrast imaging (LSCI) images of the defects collected on days 3 and 7. The scale bar represents a 20 µm scale. The scale bar represents a 20 µm scale. The amounts of the b) Col I, c) VEGF, and d) CD31 expressed in the skin tissues of the diabetic chronic wounds. The intensities of the fluorescent signals in the wounds in the control groups were set to 100% (***: *p* < 0.001, *n* = 3). e) The perfusion indexes of the diabetic chronic wounds in different groups (**: *p* < 0.01, ***: *p* < 0.001, *n* = 3). f) The mechanism for the repair of diabetic wounds using the combination of ES therapy and PHTB(TA‐siRNA) hydrogels.

Type I collagen (Col I), which promotes cellular proliferation, migration, and differentiation, is the support structure of the skin.^[^ ^37^ ^]^ **Figure** **9**a,b shows that among the diabetic chronic wounds in the different groups, the wounds in the ES therapy‐PHTB(TA‐siRNA) hydrogel groups have the highest levels of Col I, which indicates that the combination therapy promotes collagen production. Significant amounts of Col I are detected in fibroblasts, which were labeled with vimentin. Both the TA and HLC in PHTB(TA‐siRNA) hydrogels can promote fibroblast proliferation. Moreover, the HLC can provide nutrients such as amino acids to fibroblasts to promote collagen production. The high levels of Col1 in the diabetic chronic wounds in the ES therapy‐PHTB(TA‐siRNA) hydrogel groups. We hypothesize that there are several mechanisms for the high collagen expression under combined treatment: ES promotes the degradation of hydrogels and thus the release of HLC, TA, and siRNA (MMP‐9). The siRNA (MMP‐9) contributes to decrease the degradation of collagen. Additionally, HLC and TA can promote cell proliferation and collagen deposition. Therefore, this combination therapy effectively promotes the production of collagen.

The formation of new blood vessels in diabetic wounds is necessary for an adequate exchange of oxygen and nutrients. Therefore, impaired angiogenesis delays the healing of the wounds.^[^ ^38^ ^]^ To investigate whether the combination of ES therapy and PHTB(TA‐siRNA) hydrogels can promote vascularization in diabetic wounds, we evaluated the flow of blood and formation of new blood vessels in the diabetic chronic wounds.

We used immunofluorescent staining to detect the amounts of vascular endothelial growth factor (VEGF), CD31 (a marker of the vascular endothelium), and *α*‐SMA (a marker of smooth muscle cells). The results are shown in Figure 9a,c,d. M2 macrophages, fibroblasts, and vascular endothelial cells can express VEGF, which can induce angiogenesis and improve the supply of oxygen and nutrients to accelerate wound healing.^[^ ^39^ ^]^ The diabetic chronic wounds in the ES therapy‐PHTB(TA‐siRNA) hydrogel groups show the highest amounts of VEGF among the diabetic chronic wounds in the different groups. On the other hand, the diabetic chronic wounds in the ES therapy groups exhibit some amounts of VEGF, which suggests that ES therapy can promote VEGF production and vascularization. In addition, the diabetic chronic wounds in the ES therapy‐PHTB(TA‐siRNA) hydrogel groups show the highest amounts of CD31 and the highest number of blood vessels among the diabetic chronic wounds in the different groups. Similarly, the highest VEGF levels and number of blood vessels in the burns in the diabetic rats in the ES therapy‐PHTB(TA‐siRNA) hydrogel group in ES therapy‐PHTB(TA‐siRNA) hydrogel groups among the burn wounds in the different groups (Figure S9, Supporting Information). These results suggest that the combination of ES therapy and PHTB(TA‐siRNA) hydrogels improves nutrient transportation and promotes epithelialization and tissue remodeling. We hypothesized that the production of pro‐angiogenic factors and proliferation of endothelial cells, both of which are promoted by the polarization of M1 macrophages to M2 macrophages, and the secretion of the growth factors that facilitate angiogenesis (such as VEGF) are the mechanisms for the vascularization‐promoting effect of the combination of ES therapy and PHTB(TA‐siRNA) hydrogels. In this study, we showed that the combination therapy significantly enhanced blood flow in regenerating tissues at the early stage of wound healing, which indicates that the therapy accelerates the formation and maturation of blood vessels.

As shown in Figure 9a,e, on day 3, the perfusion indexes of the diabetic chronic wounds in the control group and commercial dressing group are lower than those of the diabetic chronic wounds in the PHTB hydrogel group, PHTB(TA‐siRNA) hydrogel group, ES therapy‐PHTB hydrogel group, and ES therapy‐PHTB(TA‐siRNA) hydrogel group; the perfusion index of the wounds in the diabetic rats in the ES therapy‐PHTB(TA‐siRNA) hydrogel group is the highest. On day 7, the perfusion indexes of the diabetic chronic wounds in the control group and commercial dressing group increase, which indicates the repair of the wounds. However, the perfusion index of the full‐thickness skin defects in the diabetic rats in the ES therapy‐PHTB(TA‐siRNA) hydrogel group decreases due to the contraction of the wounds and growth of epidermis in the wounds, which results in the inability of LSCI to penetrate the epidermis of the rats. The burns in the diabetic rats in the ES therapy‐PHTB(TA‐siRNA) hydrogel group show the highest perfusion index among the burns in the diabetic rats in the different groups (Figure S9, Supporting Information). Thus, the combination of ES therapy and PHTB(TA‐siRNA) hydrogels promotes vascularization in the diabetic chronic wounds.

To evaluate the toxicity of PHTB(TA‐siRNA) hydrogels in vivo, the major organs (hearts, livers, kidneys, lungs, and spleens) of the diabetic rats with the diabetic chronic wounds were stained with H&E staining. As shown in Figure S10 (Supporting Information), there is negligible damage to the organs derived from the diabetic rats in the PHTB(TA‐siRNA) hydrogel group and ES therapy‐PHTB(TA‐siRNA) hydrogel group. This confirms that PHTB(TA‐siRNA) hydrogels are wound dressings with no harmful health effect.

In Figure 9f, we summarized the mechanism for the repair of diabetic wounds using the combination of ES therapy and PHTB(TA‐siRNA) hydrogels. Commonly, diabetic chronic wounds exhibit excessive levels of proinflammatory factors and ROS but insufficient degrees of angiogenesis and collagen formation.^[^ ^40^ ^]^ Persistent inflammation may impair the regulatory effects of the immune system, which leads to the failure to repair wounds.^[^ ^41^ ^]^ An excess amount of ROS disrupts cellular homeostasis, and large amounts of MMPs damage the ECM and cell membrane, which leads to severe endothelial‐cell damage and premature cell failure.^[^ ^42^ ^]^ Therefore, biomaterials that can modulate the responses of the immune and inflammatory systems, inhibit the overproduction of MMP‐9 and ROS, and promote the formation of blood vessels and collagen can promote the healing of chronic wounds. In wounds, the conduction of the electric current generated by the natural endogenous “battery” of the epidermis is blocked. Therefore, we constructed adaptive, conductive PHTB(TA‐siRNA) hydrogels and used them in combination with ES therapy to treat diabetic chronic wounds.

We prepared a self‐assembled nanogel carrying siRNA, formed by TA and siRNA self‐assembly through hydrogen bonding, which can effectively promote cellular uptake and efficiently facilitate gene silencing. We attributed the facilitated cellular uptake of the nanogels to the binding of the phenolic groups of TA to proteins on the cell membrane. TA contains many hydrolyzable ester bonds, and the hydrolysis of the ester bonds can cause the network structure of TA‐siRNA nanogels to collapse and release siRNA, which silences the target gene, and gallic acid, which is the hydrolysis product of TA. We introduced this nanogel into the original PHTB hydrogel, so as to prepare a PHTB(TA‐siRNA) hydrogel in combination with ES for the treatment of diabetic chronic wounds. PHTB (TA‐siRNA) hydrogel is ROS‐responsive and can effectively scavenge ROS. We found that the effects of hydrogel and ES on diabetic wounds are actually interactive. The cellular uptake of TA‐siRNA could be increased by ES. The PHTB (TA‐siRNA) hydrogel was placed over the wound, where the content of siRNA was increased with ES applied, in comparison with that in the absence of ES. These results suggest that ES promotes the release of nanogels in hydrogels as well as their cellular internalization. We hypothesized that ES affected the movement of charged nanogels to promote the release of charged nanogels from the hydrogel. ES also promoted blood circulation and tissue fluid flow in biological organisms, which aided the rapid degradation of hydrogels, accelerating the release of TA‐siRNA nanogels. Therefore, ES enhances the release of siRNA from PHTB(TA‐siRNA) hydrogels and cellular uptake of the siRNA. Furthermore, ES can also promote the motion of charged TA‐siRNA nanogels, facilitating their binding with proteins on cell membranes through the phenolic hydroxyl groups of TA, and this anchoring to cell membranes can promote the celluar uptake of nanogels. We believed that when ES was used alone or combined with fixed shape wound dressing, the current could not reach the deep area of wounds, resulting in poor efficacy of ES. Therefore, we designed an adaptive conductive hydrogel, which had good flowability to fill deep areas and complex geometries, and this hydrogel acted as a conductive media to efficiently transmit electrical currents and thus exerted the therapeutic effects of ES. In this study, we loaded TA‐siRNA nanogels into this adaptive conductive hydrogel and this adaptivity not only facilitated current transport in deep wounds, but also promoted the release of TA‐siRNA nanogels in deep wounds.

In diabetic chronic wounds, PHTB(TA‐siRNA) hydrogels silenced the *MMP9* gene and thus reduced the degradation of collagen and damage to cellular structures. TA, which showed good anti‐inflammatory and antioxidant properties, effectively scavenged ROS. On the other hand, HLC, which showed some anti‐inflammatory effects, promoted fibroblast proliferation and collagen production. ES therapy‐PHTB(TA‐siRNA) hydrogel regulated the polarization of M1 macrophages to M2 macrophages. The M2 macrophages secreted anti‐inflammatory and proregenerative factors, which promoted the transition of the inflammatory phase into the proliferative phase, reduced inflammation, and promoted vascularization. Compared to the control group, the rate of wound healing was significantly increased and inflammation responses were downregulated in the ES group, with lower level of MMP‐9, TNF‐*α*, and IL‐6, higher level of angiogenic factor VEGF and higher perfusion, indicating that ES treatment could have a beneficial effect on accelerating wound healing. In ES therapy‐PHTB(TA‐siRNA) hydrogel group, ES and adaptive hydrogel dressings provided separate therapeutic effects on the wound, and the combination of the two treatment strategies could amplify these effects. Concretely, the hydrogel acted as an electrically conductive media that allowed the transmission of electrical currents to the deep areas of the wound, thus promoting the efficacy of ES. Meanwhile, ES promoted the release of nanogels from the hydrogel and the celluar internalization of nanogel. The combination of ES therapy and PHTB(TA‐siRNA) hydrogels reduced the time‐to‐repair of full‐thickness skin defects and burns in diabetic rats by 47.4% and 39.4%, respectively. Therefore, the combination of ES therapy and PHTB(TA‐siRNA) hydrogels accelerated the healing of diabetic chronic wounds.

## 3 Conclusion

In this work, self‐assembled TA‐siRNA nanogels, which effectively carried siRNA to cells and released the siRNA to silence the *MMP9* gene, were prepared. The nanogels were mixed with PVA, HLC, TA, and borax to form adaptive, conductive PHTB(TA‐siRNA) hydrogels, which were cross‐linked by dynamic borate ester bonds and hydrogen bonds. The hydrogels were able to fill deep irregular wounds and release drugs or genes into the wounds. Moreover, the hydrogels improved the transmission of the electric current, thus, promoted intercellular signaling and improved the efficacy of ES therapy. Furthermore, in response to high ROS levels in chronic wounds, the borate ester bonds in the hydrogels were oxidized and thus broken, and TA‐siRNA nanogels were released. The nanogels released siRNA, which silenced the *MMP9* gene, and TA, which inhibited inflammation and reduced the ROS level. ES therapy promoted the in vivo release of TA‐siRNA nanogels from PHTB(TA‐siRNA) hydrogels and endocytosis of the nanogels. The combination of ES therapy and PHTB(TA‐siRNA) hydrogels significantly reduced the levels of MMP‐9, ROS, and pro‐inflammatory factors. Moreover, the combination therapy promoted macrophage polarization, collagen production, and blood‐vessel formation in the diabetic chronic wounds. This work provides a promising effective strategy to construct gene‐delivery systems that promote the repair of diabetic chronic wounds. The results of this work encourage further research on effective therapies for complex chronic wounds. In the future, we will further develop piezoelectric adaptive hydrogels, enabling energy self‐supply with no need for external ES and providing a convenient treatment strategy for clinical translation.

## 4 Experimental Section

### Materials

PVA (*M*_r_ = 89000–98000) was purchased from Sigma–Aldrich. TA (*M*_r_ = 1700) was purchased from Aladdin. HLC (China patent number: ZL01106757.8, *M*_r_ = 97000) was purchased from Xi'an JUZI Biology Gene Technology Co., Ltd. siRNA was supplied by GenePharma as shown in Table S1 (Supporting Information). All other reagents are of analytical grade without special treatment. All animal experiments were reviewed by the Northwest University Laboratory Animal Management and Ethics Committee. The experiments complied with the legal and institutional guidelines related to animal ethics and were approved by the Ethics Committee (NWU‐AWC‐20210810R).

### Preparation of Self‐Assembled TA‐siRNA Nanogels

An siRNA solution (0.2 optical density (OD) mL^−1^) and a TA solution (0.2 wt%) were prepared in nuclease‐free phosphate‐buffered saline (PBS). One milliliter of the siRNA solution was placed in an ice‐water bath and stirred at 100 rpm on a magnetic stirrer. Then, within 3 min, 1 mL of the TA solution was added dropwise to the siRNA solution, and the mixture was reacted for 10–30 min. The resulting TA‐siRNA nanogels were passed through a centrifugal filtration device (Amicon Ultra‐4 Ultracel‐100K, Millipore), whose molecular weight cutoff is 100 kDa, to remove unreacted TA and siRNA. Then, 200 µL of RNase‐free PBS was added to the nanogels, and the stock solution of TA‐siRNA nanogels was stored at 4 °C.

### Characterization of TA‐siRNA Nanogels

SEM and TEM were used to observe the morphology of TA‐siRNA nanogels. The size distribution and zeta potential of the nanogels were measured using a dynamic light scattering analyzer (Zetasizer Nano ZS, Malvern). The gel fraction of the nanogels was determined on the basis of the weight method. The detailed procedures for characterizing the nanogels are available in Supporting Information.

### Cellular Internalization and Gene‐Silencing Efficiency of TA‐siRNA Nanogels

RAW264.7 macrophages were cultured in Dulbecco's modified Eagle medium (DMEM). A sterilized coverslip was placed on a six‐well plate, and viable cells (2 × 10^5^ cells per well) were seeded into the plate. Two milliliters of fresh DMEM were added to the cells, and the cells had been incubated for 6 h to allow for cellular apposition before being used for cellular internalization and gene‐silencing experiments. The TA‐siRNA nanogels in the stock solution of TA‐siRNA nanogels were prepared under sterile conditions, and the siRNA used for preparing the nanogels was labeled with 5‐carboxyfluorescein (5‐FAM). To measure the cellular internalization of TA‐siRNA nanogels, an siRNA solution (100 µL per well, 1 OD mL^−1^) or the stock solution of TA‐siRNA nanogels (100 µL per well) to the RAW264.7 cells in DMEM was added, and the cells had been incubated in a CO_2_ incubator before being collected at different intervals (2, 6, or 12 h). The cells were washed with PBS and stained with 4,6‐diamidino‐2‐phenylindole (DAPI). Then, the cells were observed under a confocal microscope (Nikon‐A1, Japan), and the intensities of the fluorescent signals generated in the cells were quantitatively analyzed using ImageJ software. The cell culture medium was also collected, and the content of residual 5‐FAM‐labeled siRNA in the culture medium was measured using Fluorescence multimode microplate reader (INFINITE M PLEX, Tecan), and the cellular uptake rate of the siRNA was calculated according to Equation 1:

(1) $$\mathrm{Cellular}\;\mathrm{uptake}\;\mathrm{rate}=100\times \left({\mathrm{FI}}_{\mathrm{initial}}-{\mathrm{FI}}_{\mathrm{residual}}\right)/{\mathrm{FI}}_{\mathrm{initial}}$$

where FI_initial_ is the initial fluorescent intensity of 5‐FAM‐labeled siRNA, and FI_residual_ is the residual fluorescent intensity of 5‐FAM‐labeled siRNA in the culture medium. To measure the gene‐silencing efficiency of TA‐siRNA nanogels, LPS (1 µg mL^−1^) was added to the RAW264.7 cells in DMEM to increase the level of MMP‐9 in the cells. After cell induction for 12 h, the solution of siRNA(MMP‐9) (100 µL per well, 1 OD mL^−1^) or the stock solution of TA‐siRNA(MMP‐9) nanogels containing the siRNA(MMP‐9) gene was added into the cells. An siRNA(N.C.) solution (100 µL per well, 1 OD mL^−1^) or the stock solution of TA‐siRNA(N.C.) nanogels was added to the cells in the negative control groups. The negative control siRNA(N.C.) was unable to silence the *MMP9* gene. The TA‐siRNA nanogels in the stock solutions of TA‐siRNA nanogels were prepared under aseptic conditions, and the siRNA used for preparing the nanogels was labeled with 5‐FAM. Then, the cells had been incubated in a CO_2_ incubator for 12 h before being immunofluorescently stained for the MMP‐9 (bs‐0397R, Bioss). In order to distinguish it from the green fluorescent siRNA, the immunofluorescently stained secondary antibody was used with Cy3. The nucleus of the cells was labeled with DAPI, photographed using a confocal microscope, and analyzed using ImageJ software for fluorescent‐signal intensities.

### Preparation of Adaptive, Conductive PHTB(TA‐siRNA) Hydrogels

Adaptive, conductive PHTB(TA‐siRNA) hydrogels were prepared on the basis of the previous study.^[^ ^11^ ^]^ Briefly, 10 mL of a 10 wt% PVA solution was mixed with 2 mL of a 15 wt% HLC solution to prepare mixture A. Then, 4 mL of a 5 wt% borax solution, 2 mL of a 10 wt% TA solution, and 2 mL of the stock solution of TA‐siRNA nanogels were mixed to prepare mixture B. Mixtures A and B were mixed and stirred to obtain adaptive, conductive PHTB(TA‐siRNA) hydrogels. PHTB hydrogels were prepared by replacing the stock solution of TA‐siRNA nanogels with water. PB hydrogels were prepared by using the PVA solution and the borax solution and replacing the remaining components with pure water. PHB hydrogels were prepared by using the PVA solution, the HLC solution, and the borax solution and replacing the remaining components with pure water.

### Characterization of PHTB(TA‐siRNA) Hydrogels

The dynamic rheological behavior of PHTB(TA‐siRNA) hydrogels was analyzed at 25 °C using a rheometer. The detailed experimental methods are available in Supporting Information. PHTB(TA‐siRNA) hydrogels (3.0 ± 0.3 mL) were added to a 5 mL syringe to which a 20‐gauge needle, a 22‐gauge needle, or a 23‐gauge needle was attached. The length of the syringe was 50 mm. As the needle pointed down, the leakage of the hydrogels out of the needle was observed and photographed. A glass slot (10 cm height, 8 cm width, and 8 mm inner layer thickness) was filled with glass spheres whose diameter was 6 mm, and the hydrogels were placed onto the spheres. The flow of the hydrogels was observed and photographed. To examine the self‐healing properties of PHTB(TA‐siRNA) hydrogels, the hydrogels were injected, which were stained with RhB or alcian blue, into molds. The ability of the hydrogels to adapt their shape to the molds was observed and photographed. The conductivity of the hydrogels was measured according to the method reported in a previous study,^[^ ^11^ ^]^ and the detailed experimental methods are available in Supporting Information.

### In Vivo Release of Drugs from PHTB Hydrogels

Adaptive PHTB hydrogels and conventional PVA hydrogels were loaded with RhB, which served as a model drug, and the in vivo release of RhB from the hydrogels was observed. A 10% PVA solution was dissolved at 90 °C and 2 mg mL^−1^ of RhB was added to the solution. The solution was frozen at ‐20 °C for 8 h and stored at room temperature for 8 h. The conventional shape‐fixed PVA hydrogels were obtained by freezing and thawing the PVA solution three times. The adaptive PHTB hydrogels loaded with RhB were prepared similarly to PHTB(TA‐siRNA) hydrogels, except the stock solution of TA‐siRNA nanogels was replaced with a 4 wt% RhB solution. Sprague Dawley (SD) rats were anesthetized, and the hairs on the backs of the rats were removed. Two wounds that were 2 cm apart were established on the backs of the rats using a punch whose diameter is 6 mm. The adaptive PHTB hydrogels loaded with RhB or conventional PVA hydrogels loaded with RhB were applied to the two wounds and removed after 30 min. After the changes in the color of the wounds had been photographed and recorded, the rats were euthanized, and skin tissues had been taken from the wounds for paraffin embedding and sectioning before being used for microscopic observation.

### Release of Drugs in Response to ROS Levels

H_2_O_2_ solutions (0 × 10^−6^, 10 × 10^−6^, 100 × 10^−6^, and 1000 × 10^−6^ m) were prepared, and 2 mL of PHTB(TA‐siRNA) hydrogels was placed in a sample bottle. Then, 1 mL of the H_2_O_2_ solution (0 × 10^−6^, 10 × 10^−6^, 100 × 10^−6^, or 1000 × 10^−6^ m) was added to the sample bottle, and the changes in the hydrogels were observed and photographed. After 3 h, the liquid in the bottle was aspirated, and the hydrogels had been freeze‐dried under vacuum conditions before being observed using a scanning electron microscope. On the basis of the SEM images, the pore size distribution of the hydrogels was evaluated using Adobe Photoshop CC 2018. From each SEM image, 300 pore sizes were collected, and statistical data were collected to evaluate the pore size distribution of the hydrogels. To study the effects of an H_2_O_2_ environment on the release of siRNA from PHTB(TA‐siRNA) hydrogels, 2 mL of the hydrogels was added to each of 90 sample bottles. The sample bottles were divided equally into three groups. On the basis of the group to which each sample bottle belonged, a 0 × 10^−6^ m H_2_O_2_ solution, a 10 × 10^−6^ m H_2_O_2_ solution, or a 1000 × 10^−6^ m H_2_O_2_ solution was added to the bottle. Every hour, 3 out of the 30 bottles in each group were sampled, and the liquids in the bottles had been collected and refrigerated before being analyzed using agarose gel electrophoresis. To perform the agarose gel electrophoresis analysis, 10× Tris/borate/EDTA (TBE) buffer (Solarbio) was diluted to 1× TBE buffer and the 1× TBE buffer was used to configure a 5% agarose solution. The solution was heated to dissolve the agarose fully, and a nucleic acid dye (BBI Life Sciences) was added to the solution. The solution was stirred and slowly poured into a horizontal electrophoresis tank to avoid the production of bubbles. The thickness of the agarose gel was 3–5 mm, and the gel was cooled for 30 min. Twenty microliters of each sample were mixed with two microliters of a loading buffer (Takara). Then, the agarose gel was covered with the 1× TBE buffer, and 10 µL of each sample and 10 µL of a DNA ladder (O'RangeRuler DNA Ladders, Thermo Scientific) were added to the sample wells of the gel. The voltage was 30 V m^−1^. After 1 h, the power was turned off, and the gel was removed and viewed under a UV transilluminator. Quantification of siRNA was accomplished using a fluorescence‐based RNA quantification kit (RediPlate 96 RiboGreen RNA Quantitation Kit). The RiboGreen fluorescent dye binds to the RNA in a sample and produces a fluorescent signal whose intensity is proportional to the RNA content of the sample. The intensity of the fluorescent signal produced by each sample was measured using a fluorescence spectrophotometer and used to quantify the siRNA content of the sample.

### Antioxidant Properties of PHTB(TA‐siRNA) Hydrogels

Antioxidant properties of PHTB(TA‐siRNA) hydrogels were measured using in vitro and in vivo antioxidant assays. The in vitro antioxidant assays included the DPPH radical scavenging assay,^[^ ^43^ ^]^ ABTS·^+^ radical scavenging assay,^[^ ^44^ ^]^ and H_2_O_2_ scavenging assay.^[^ ^45^ ^]^ The detailed procedures for performing the antioxidant assays are available in Supporting Information. On the other hand, the in vivo antioxidant assays were performed by measuring the ability of the hydrogels to scavenge ROS. To perform the in vivo antioxidant assays, SD rats (250 g) were anesthetized and their backs were depilated. A triangle was outlined on the back of each rat, and LPS, PHTB(TA‐siRNA) hydrogels, or LPS and PHTB(TA‐siRNA) hydrogels were injected into the top of the triangle. The PHTB(TA‐siRNA) hydrogels were prepared under aseptic conditions. The rats that were injected with LPS and PHTB(TA‐siRNA) hydrogels had been injected with 50 µL of an LPS solution (10 mg mL^−1^) 2 h before being injected with 0.1 mL of PHTB(TA‐siRNA) hydrogels.^[^ ^46^ ^]^ After 24 and 72 h, respectively, skin tissues were collected from the injection site for cryosectioning and DHE staining. The ROS content of the skin tissues was determined using an ROS assay kit.

### Roles of ES Therapy in Cellular Internalization of TA‐siRNA Nanogels

A device for performing ES therapy on cells was prepared according to the previous study.^[^ ^11^ ^]^ Briefly, conductive glass was placed in a six‐well plate, and platinum wires were attached to both sides of the glass. The wires were connected to an external electrical stimulator. RAW264.7 macrophages, which were cultured in DMEM, were inoculated onto the conductive glass at a concentration of 2 × 10^5^ cells per well and added with 2 mL of fresh DMEM. The cells were incubated for 6 h to ensure cell apposition, and 100 µL per well of the stock solution of TA‐siRNA nanogels was added to the cells. Then, the cells were incubated in a CO_2_ incubator for 1 h. The cells in the ES therapy group were subjected to ES therapy (1 Hz, 0.05 mA, 30 min; polarity switching after 15 min) using an electrostimulator (spastic muscle low‐frequency therapy instrument KX‐3C, Beijing Yaoyang Kangda Medical Instrument Co., China). The cells remained incubated for 30 min after the therapy to allow for cell stabilization. The cell culture medium was collected, and the content of residual 5‐FAM‐labeled siRNA in the culture medium was measured using fluorescence multimode microplate reader (INFINITE M PLEX, Tecan), and the cellular uptake rate of the siRNA was calculated according to Equation 1. The cells were also collected by digestion and centrifugation, and a TEM fixative was added to the cells. After being incubated for 24 h, the cells were washed with distilled water, dehydrated with ethanol, and added to epoxy resin. The cell‐embedded epoxy resin was cut into thin sections with a microtome, placed on copper TEM grids, and stained with uranyl acetate and lead citrate. The morphology of the cells was observed using a transmission electron microscope.

### Roles of ES Therapy in the Release of siRNA from PHTB(TA‐siRNA) Hydrogels In Vivo

SD rats were anesthetized, and wounds were created on their backs using a punch whose diameter is 6 mm. PHTB(TA‐siRNA) hydrogels, whose siRNA was labeled with 5‐FAM, were applied to the wounds. After 30 min, ES therapy was performed on the wounds for 30 min using an 8 mA pulsed current whose frequency was 1 Hz. The polarity of the current was switched after 15 min. Then, skin tissues were taken from the wounds for cryosectioning, and the tissues were stained with DAPI for cell nuclear labeling. The stained tissues were observed under a confocal microscope.

### Diabetic Rats

Rats whose blood glucose levels were greater than or equal to 16.7 mmol L^−1^ after being intraperitoneally injected with a 1% citrate‐buffered streptozotocin solution were selected as diabetic rats,^[^ ^47^ ^]^ and the detailed experimental methods are available in Supporting Information.

### Repairs to Full‐Thickness Skin Defects in Diabetic Rats

The procedures for assessing the repair of the full‐thickness skin defects in the backs of the diabetic rats to which the combination of ES therapy and PHTB(TA‐siRNA) hydrogels was applied are shown Figure S9 (Supporting Information). The experimental groups were: blank control group, DuoDERM group, PHTB hydrogel group, PHTB(TA‐siRNA) hydrogel group, ES therapy group, ES therapy‐PHTB hydrogel group, and ES therapy‐PHTB(TA‐siRNA) hydrogel group. The frequency and intensity of the pulsed current were 1 Hz and 8 mA, respectively, and the current was applied for 10 d. The polarity of the current was switched on day 4. The detailed experimental procedures are available in Supporting Information.

### Repairs to Burns in Diabetic Rats

Deep second‐degree burns were established in the backs of the diabetic rats using a temperature‐controlled tabletop scalding instrument.^[^ ^48^ ^]^ The experimental groups were the blank control group, PHTB(TA‐siRNA) hydrogel group, and ES therapy‐PHTB(TA‐siRNA) hydrogel group. The detailed experimental procedures are available in Supporting Information.

### Histological Analysis of Repairs to Full‐Thickness Skin Defects and Burns in Diabetic Rats

The diabetic rats were euthanized, and skin tissues were removed from the full‐thickness skin defects and burns of the rats. The tissues were fixed in neutral formaldehyde for 1 d. Then, the tissues had been embedded in paraffin and sectioned before being subjected to H&E, DHE, and immunofluorescent staining. Immunofluorescence single staining: VEGF (bs‐1313R, Bioss), CD86 (13395‐1‐AP, Proteintech), CD206 (60143‐1, Proteintech), TNF‐*α* (bs‐10802R, Bioss), HIF‐1*α* (bs‐1407R, Bioss), MMP‐9 (bs‐4593R, Bioss), IL‐6 (bs‐0782R, Bioss) and IL‐10 (bs‐6761R, Bioss). Immunofluorescence double staining: *α*‐SMA (bsm‐33188 M, Bioss)/CD31 (GB11063‐2, Servicebio) and Col I (14695‐1‐AP, Proteintech)/Vimentin (GB12192, Servicebio)). The cell nucleus of the tissues were labeled with DAPI, and the photographs were analyzed for intensities of fluorescent signals using ImageJ software. The intensities of the fluorescent signals in the full‐thickness skin defects and burns of the diabetic rats in the control groups were set to 100%. Laser Speckle Contrast Imaging (RFLSI Pro) was used to examine blood perfusion in the full thickness skin defects and burns.

### Statistical Analysis

All data in our study were derived from at least three independent experiments. The SPSS software was used for statistical analyses. The above experimental data were analyzed by the one‐way ANOVA with Tukey’ post hoc test and expressed as means ± standard deviations. Student's t‐tests were used to determine if the variance between groups was similar or between only two groups. Statistical significance was defined as having *, *p* < 0.05; **, *p* < 0.01; ***, *p* < 0.001.

## Conflict of Interest

The authors declare no conflict of interest.

## Supporting information

Supporting Information

## Data Availability

Research data are not shared.
