# Supplementary material for: A Combination Therapy Using Electrical Stimulation and Adaptive, Conductive Hydrogels Loaded with Self‐Assembled Nanogels Incorporating Short Interfering RNA Promotes the Repair of Diabetic Chronic Wounds
Source: Adv Sci (Weinh). 2022 Sep 5;9(30):2201425. doi: 10.1002/advs.202201425 (PMC9596839; doi:10.1002/advs.202201425)
Supplement: Supplementary file 1 — Supporting Information [file ADVS-9-2201425-s001.pdf]

ADVANCED SCIENCE
Open Access

Supporting Information

for *Adv. Sci.*, DOI 10.1002/advs.202201425

A Combination Therapy Using Electrical Stimulation and Adaptive, Conductive Hydrogels Loaded with Self-Assembled Nanogels Incorporating Short Interfering RNA Promotes the Repair of Diabetic Chronic Wounds

*Huan Lei and Daidi Fan**

© Copyright 2020. WILEY-VCH GmbH.

Supporting Information

A combination therapy using electrical stimulation and adaptive, conductive hydrogels loaded with self-assembled nanogels incorporating short interfering RNA promotes the repair of diabetic chronic wounds

*Huan Lei, Daidi Fan**

E-mail: fandaidi@nwu.edu.cn

Keywords: self-assembled nanogels, siRNA delivery, hydrogel-ES combination therapy, diabetic burn

Results

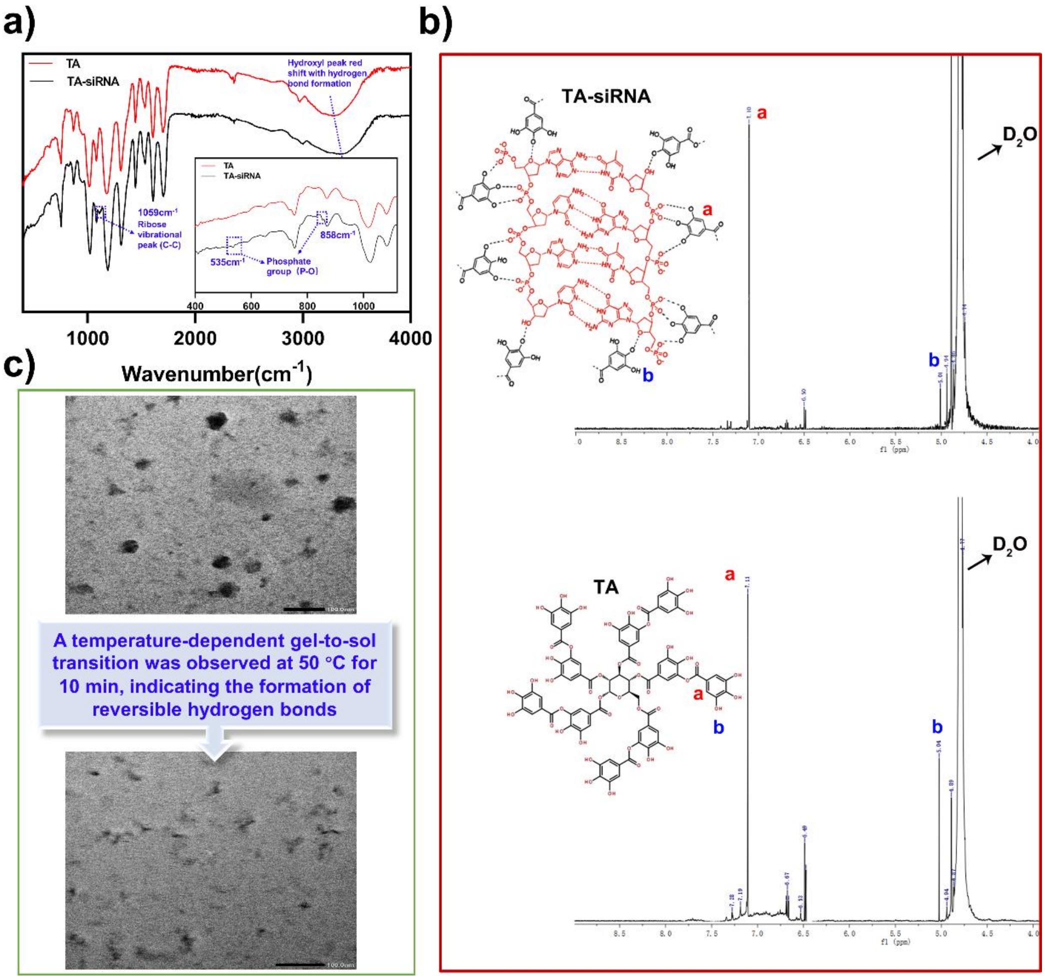

Figure S1 a) FTIR spectra of TA-siRNA nanogels, b) The 1H NMR spectra of TA-siRNA nanogels, c) Temperature-dependent gel-to-sol transition after heating to 50°C for 10 min.

The FTIR spectra in Figure S1a show that the -OH peak of TA-siRNA (3200-3700 cm^{-1}) is red-shifted compared to that of TA, implying that TA-siRNA may form hydrogen bonds. The FTIR spectrum of TA-siRNA has a vibrational peak of ribose detected at 1095.49 cm^{-1} and characteristic peaks of phosphate groups are observed at 858 cm^{-1} and 535 cm^{-1}, indicating that TA may forms hydrogen bonds with siRNA. The 1H NMR results showed that the hydrogen peak of the TA phenolic hydroxyl group in TA-siRNA (Figure S1b, δ 5.0) was significantly weaker compared to that of TA, suggesting that the hydroxyl group of TA interacts with siRNA. After heating the TA-siRNA nanogels for 10 min, the collapse of the nanogel structure was observed by TEM (Figure S1c), and this temperature-dependent gel-to-sol transition suggests that the main cross-linking mechanism of TA-siRNA nanogels is the generation of reversible hydrogen bonds.

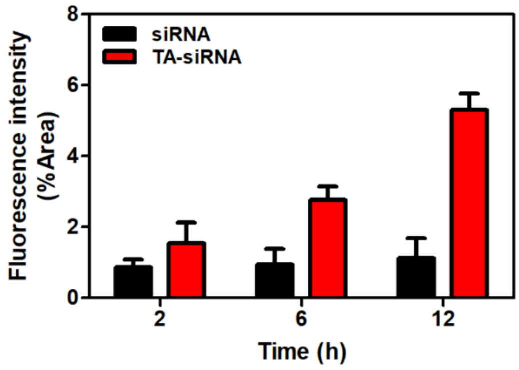

Figure S2 Quantitative fluorescence analysis of Figure 1e images with Image J software (n = 5).

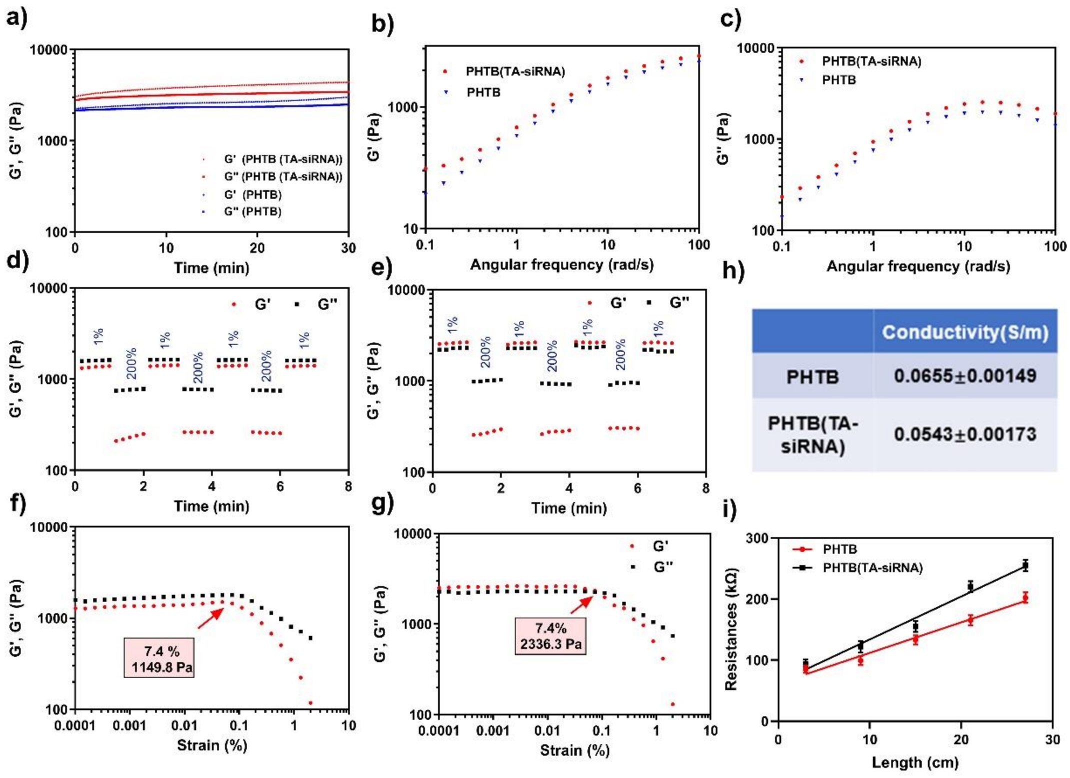

Figure S3. a) Rheodynamics: amplitude-time scan (strain 1%), b) Rheodynamics: G' for frequency scanning, c) Rheodynamics: G'' for frequency scanning, d) Amplitude-strain scans of PHTB(TA-siRNA) hydrogels, e) Amplitude-strain scans of PHTB hydrogels, f) Rheological properties of PHTB(TA-siRNA) hydrogels under alternating strain, g) Rheological properties of PHTB hydrogels under alternating strain, h) Electrical conductivity of PHTB(TA-siRNA) hydrogels (n = 5), i) resistance values of PHTB(TA-siRNA) hydrogels and PHTB hydrogels stretched at different lengths (n = 3).

We performed rheological tests on the PHTB hydrogels and the adaptive conductive hydrogels PHTB(TA-siRNA) loaded with self-assembled TA-siRNA nanogels. From

the results of the amplitude-time scan (figure S2a), both G' and G'' of PHTB (TA-siRNA) increased to a certain extent compared with PHTB, and a similar phenomenon was observed under the frequency scan (figure S2b, c), which indicates that there is a certain degree of cross-linking between the TA-siRNA nanogel and the PHTB hydrogel backbone, and this cross-linking can enhance the mechanical properties of the hydrogel in a small degree, but does not greatly affect its own morphology and mechanical characteristics. As shown in Fig. figure S2d, e, in the linear viscoelastic region G' and G'' of PHTB (TA-siRNA) hydrogel are larger than those of PHTB hydrogel G' and G' ' are larger, and the addition of nanogels leads to some increase in cross-link density in the hydrogels, while the Tan δ (G''/ G', the ratio of viscoelasticity to elasticity) of both hydrogels is within the range of 1.0 ± 0.1, indicating that they both have good self-adaptability. When Tan δ is greater than 1, it means that G'' is greater than G', and the viscosity is mainly represented as fluid; when Tan δ is less than 1, it means that G'' is less than G', and the elasticity is mainly represented as gel solid state; and when Tan δ is equal to 1, it means that G'' is equal to G', and the state of the sample is in the transition point between gel state and fluid state, which means that the gel has good self-adaptability. As the stress value increases(figure S2f, g), both hydrogels show a flow point at 7.4%, indicating that the gel structure is disrupted at this point, and the stress value of PHTB (TA-siRNA) hydrogel at the flow point (2336.3 Pa) is larger than that of PHTB hydrogel (1149.8 Pa), which is also the contribution of TA-siRNA nanogel. The alternating strain in rheometry can respond to the self-healing performance of the hydrogels, as shown in Figure figure S2f, g. After several cycles of

1% and 200% strain change, the G' and G'' values of both PHTB(TA-siRNA) hydrogels and PHTB hydrogels were able to return to their initial values when the strain was restored from 200% to 1%, indicating that both hydrogels can achieve self-healing and rebuild their structures in a very short time.

We further investigated whether the addition of TA-siRNA nanogels affects the conductivity of PHTB hydrogels, and the results are shown in figure S2h. The conductivity of PHTB (TA-siRNA) hydrogels was reduced compared to PHTB gels, which we analyzed was due to the addition of TA-siRNA nanogels, which formed borate ester bonds with PVA or TA, thus consuming some of the conductive boron ions in the system, while the increase in cross-link density might hinder the directional movement of charged ions thus reducing the conductivity of the hydrogels. Then we further investigated the resistance change of PHTB(TA-siRNA) hydrogel and PHTB gel in the stretched state, and the results are shown in Fig. figure S2i. The resistance of PHTB(TA-siRNA) hydrogel and PHTB gel in the stretched state is linearly changed, which indicates the relatively stable conductivity of the two hydrogels.

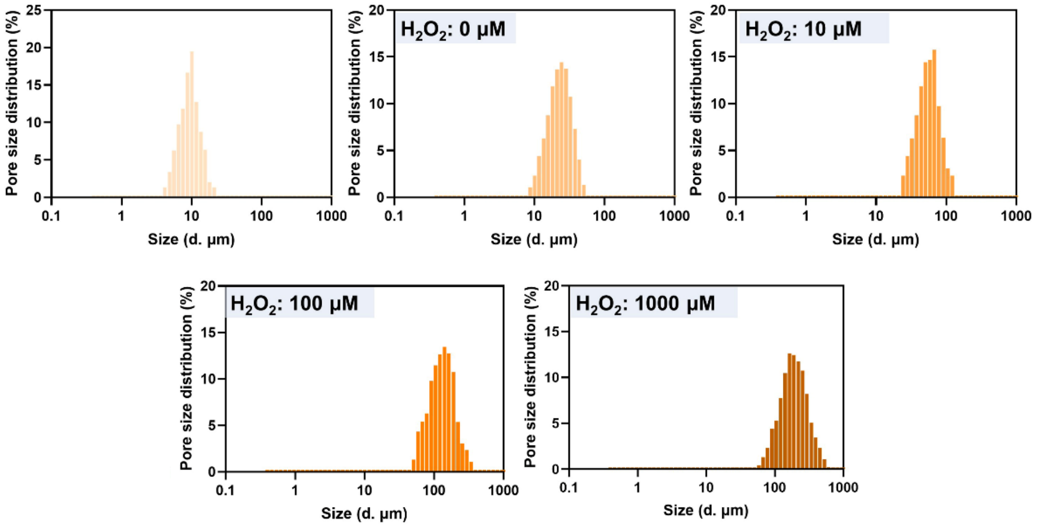

Figure S4 The pore size distribution of the hydrogels after the addition of the different concentrations of H_2O_2.

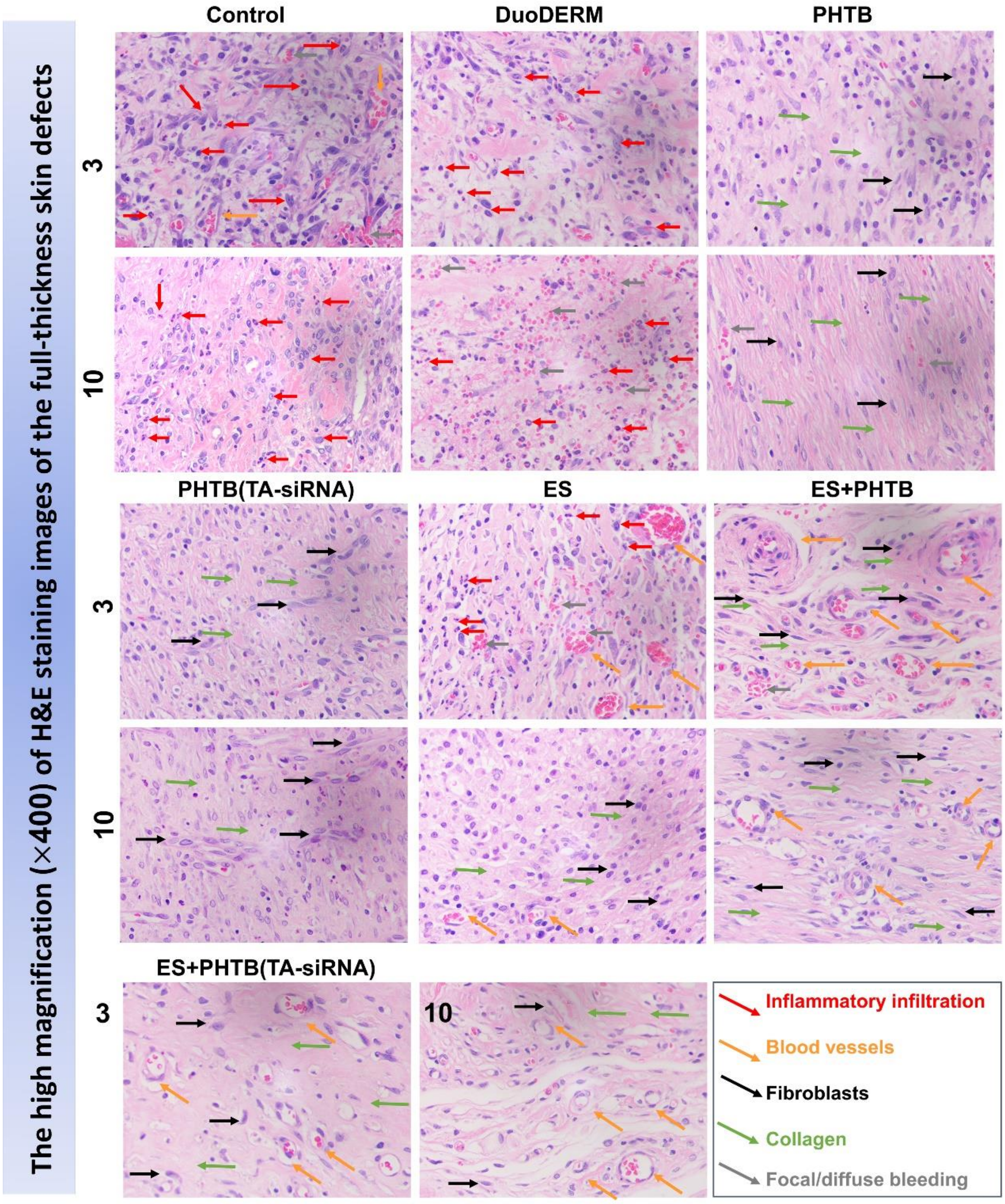

Figure S5. The high magnification (×400) of H&E staining images of the full-thickness skin defects

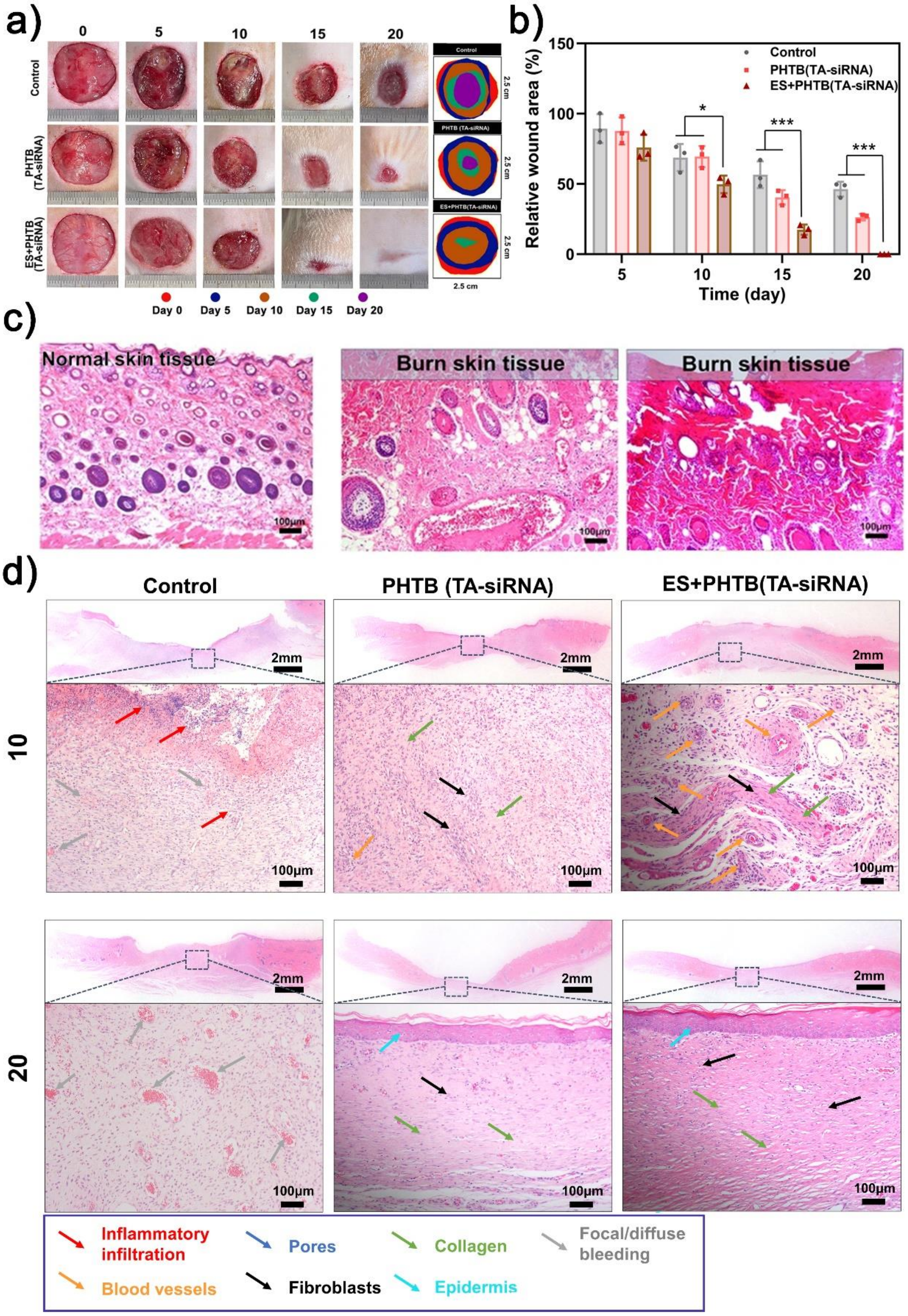

Figure S6 a) Photographs of burns in diabetic rats and the healing trajectory of the burns. b) The healing rates of the burns on days 5, 10, 15, and 20 (*: $p < 0.05$, ***: $p < 0.001$, $n = 3$). c) The H&E staining of normal skin tissues and the skin tissues with the burns. d) The low-magnification (scale bar: 2 mm) and high-magnification (scale bar: 100 μm) images of the burns on days 10 and 20.

Burns were established in the backs of diabetic rats. As shown in **Figure S6a**, on days 5 and 10, the burns in the diabetic rats in the control group show significant ulceration, which indicates the severity of the burns. Both PHTB(TA-siRNA) hydrogels and the combination of the hydrogels and ES therapy promote the healing of burns of diabetic rats. The burns in the diabetic rats in the ES therapy–PHTB(TA-siRNA) hydrogel group are completely closed on day 20, while the burns in the diabetic rats in the control group are completely closed on day 33. Therefore, the combination therapy reduces the repair time of burns in diabetic rats by 39.4%. The healing rates of burns of diabetic rats in different groups are shown in **Figure S6b.** On day 20, the areas of the burns in the diabetic rats in the control group are 46.2 ± 5.3% of the initial areas of the burns. On the other hand, the areas of the burns in the diabetic rats in the PHTB(TA-siRNA) hydrogel group are 26.4 ± 2.2% of the initial areas of the burns. In comparison, the burns in the diabetic rats in the ES therapy–PHTB(TA-siRNA) hydrogel group are completely closed, which indicates the high efficacy of the combination therapy to repair burns in diabetic rats.

The degree of the repair of burns of diabetic rats was further assessed by using H&E staining. As shown in **Figure S6c**, normal skin tissues have intact epidermis and abundant subcutaneous attachments (pores, blood vessels, collagen fibers, and fibroblasts), whereas skin tissues with burns show significant degrees of hemorrhage and edema, extensive infiltration of inflammatory cells, swelling of collagen fibers, damages to blood vessels, severe hemorrhage of subcutaneous tissues, and destruction of dermal tissues. Thus, the physiological characteristics of the burns in diabetic are more complex than those of the full-thickness skin defects in diabetic. As shown in **Figure S6d and Figure S7**, on day 10, the burns in the diabetic rats in the control group show significant degrees of inflammation, vascular trauma, and focal hemorrhage. The number of the inflammatory cells that infiltrated the burns of the diabetic rats in the PHTB(TA-siRNA) hydrogel group and ES therapy–PHTB(TA-siRNA) hydrogel group is lower than the number of the inflammatory cells that infiltrated the burns of the diabetic rats in the control group. The burns in the diabetic rats in the ES therapy–PHTB(TA-siRNA) hydrogel group show the proliferation of fibrous tissues and

formation of new capillaries. On day 20, the inflammation in the burns in the diabetic rats in the control group persists, and the infiltration of multifocal lymphocytes and formation of multinucleated giant cells are observed. Moreover, epidermal structures are absent. In contrast, there is almost no inflammation in the burns in the diabetic rats in the ES therapy–PHTB(TA-siRNA) hydrogel group, and there are few inflammatory cells infiltrating the burns. Moreover, epidermal structures are completely restored in the burns, which indicates that the combination of ES therapy and PHTB(TA-siRNA) hydrogels promotes microvascular formation, reduces inflammation, and promotes wound closure.

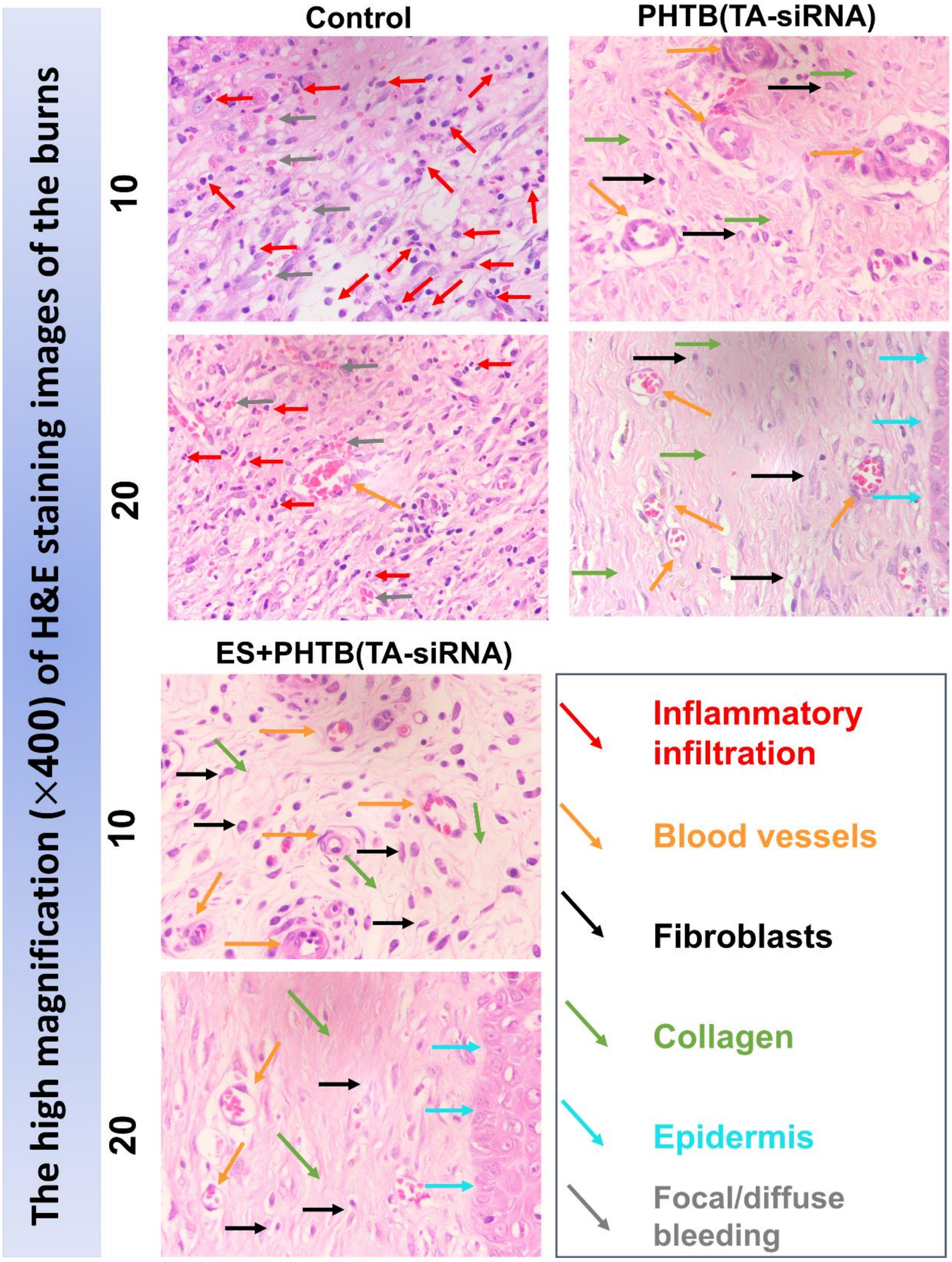

Figure S7. The high magnification (×400) of H&E staining images of the burns

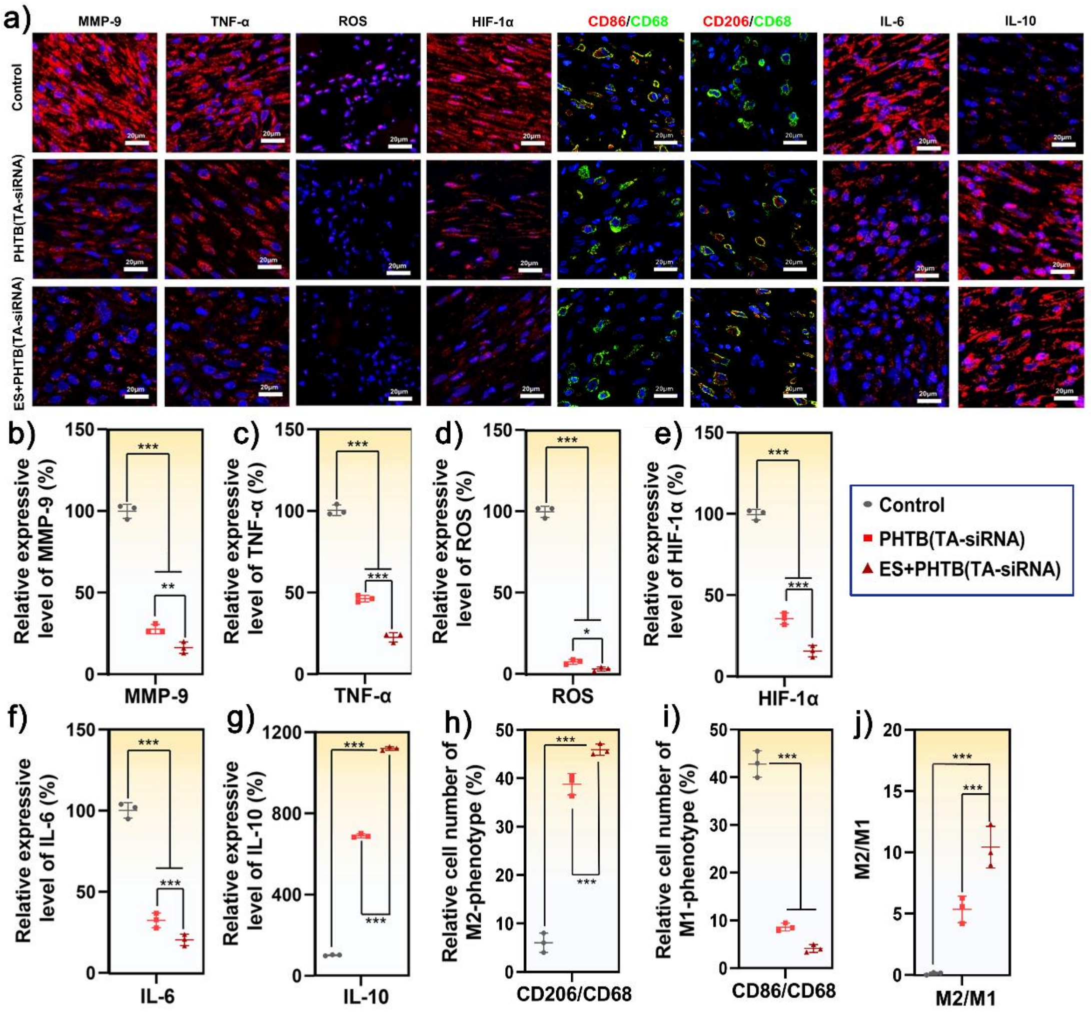

Figure S8. a) The immunofluorescent staining of the MMP-9, TNF-α, HIF-1α, IL-10, and IL-6 expressed in the skin tissues of the burns in the backs of diabetic rats and the labeling of the ROS and macrophage phenotypic markers produced in the skin tissues. The blue color represents DAPI-stained cell nucleus, and the ROS was labeled with a DHE fluorescent probe (violet color). Immunofluorescence double staining of CD68-labelled (green) macrophages, and CD206-labelled (red) M2-type macrophages. Immunofluorescence double staining of CD68-labelled (green) macrophages, and CD86-labelled (red) M1-type macrophages. The analyses were performed on day 10, and the scale bar represents a 20-μm scale. The quantitative analyses for the intensities of the fluorescent signals produced by b) MMP-9, c) TNF-α, d) ROS, e) HIF-1α, f) IL-6, and g) IL-10. The intensities of the fluorescent signals in the burns of the diabetic rats in the control groups were set to 100% (**: $p < 0.01$, ***: $p < 0.001$, $n = 3$). h) The population of double-positive M1 macrophages and i) that of double-positive M2 macrophages(***: $p < 0.001$, $n = 3$). j) The population ratios of M2 macrophages to M1 macrophages (***: $p < 0.001$, $n = 3$).

The amounts of MMP-9 in the burns in the diabetic rats in the control group are higher than those of MMP-9 in the full-thickness skin defects in the diabetic rats in the control

group (**Figure S8**a and **S8**b), which indicates the higher severity of the burns than the full-thickness skin defects. The combination of ES therapy and PHTB(TA-siRNA) hydrogels significantly reduces the amounts of MMP-9 in full-thickness skin defects and burns in diabetic rats. The amounts of ROS and HIF-1α in the burns in the diabetic rats in the control group are significantly higher than those in the full-thickness skin defects in the diabetic rats in the control group (**Figure S8**a, **S8**d and **S8**e), which indicates that the diabetic rats with burns experience a greater degree of hypoxia than do the diabetic rats with full-thickness skin defects. The treatment of the full-thickness skin defects and burns in the diabetic rats with the combination of ES therapy and PHTB(TA-siRNA) hydrogels significantly reduces the degree of hypoxia in the defects and burns. A large number of M1 macrophages were detected in the burns of the diabetic rats in the control group, and large amounts of IL-6 were detected in the burns in the diabetic rats in the control group (**Figure S8**f-j), which indicates the pro-inflammatory states of the burns. In contrast, a large number of M2 macrophages and IL-10 were detected in the burns of the diabetic rats in the ES therapy–PHTB(TA-siRNA) hydrogel group.

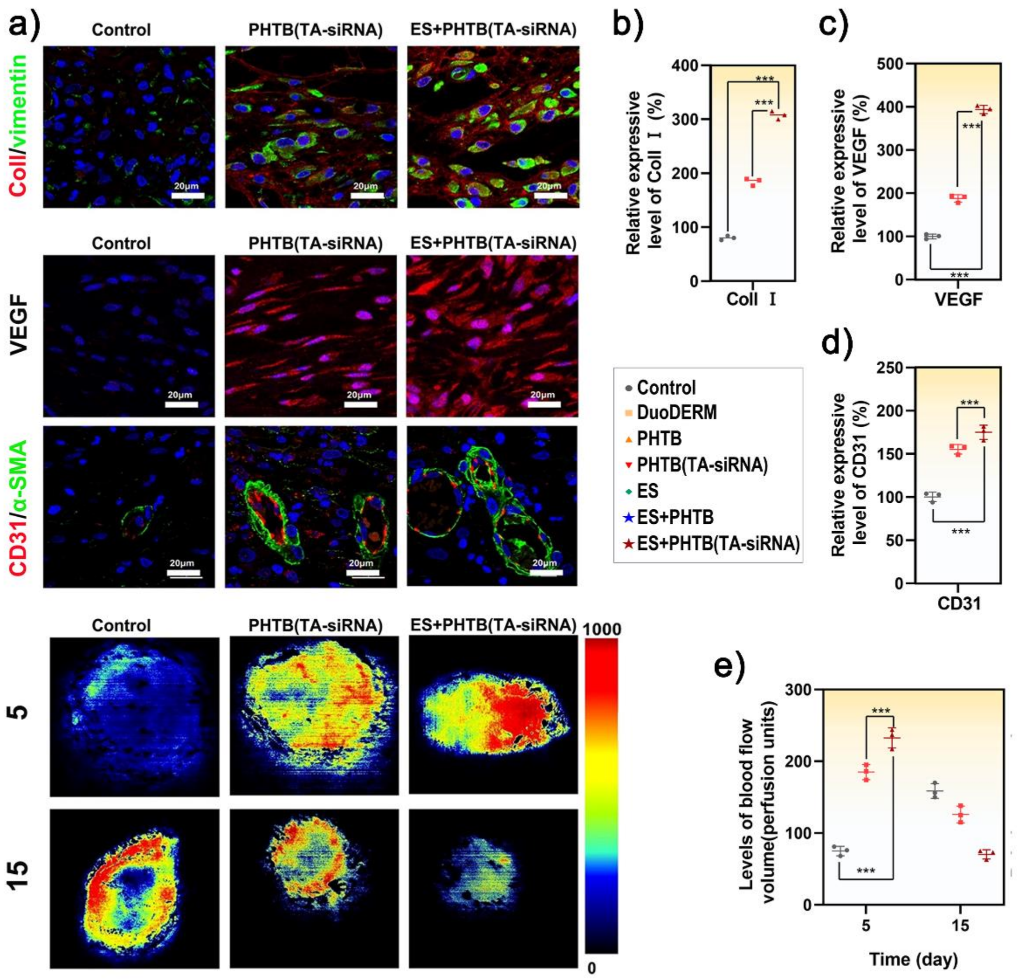

Figure S9. a) The immunofluorescent staining of the Col I, VEGF, and CD31 expressed on day 10 in the skin tissues of the burns of diabetic rats and the LSCI images of the burns collected on days 5 and 15. The scale bar represents a 20-µm scale. The amounts of the b) Col I, c) VEGF, and d) CD31 expressed in the skin tissues of the burns in diabetic rats. The intensities of the fluorescent signals in the burns of the diabetic rats in the control groups were set to 100% (***: $p < 0.001$, $n = 3$). e) The perfusion indexes of the burns of diabetic rats in different groups (**: $p < 0.01$, ***: $p < 0.001$, $n = 3$).

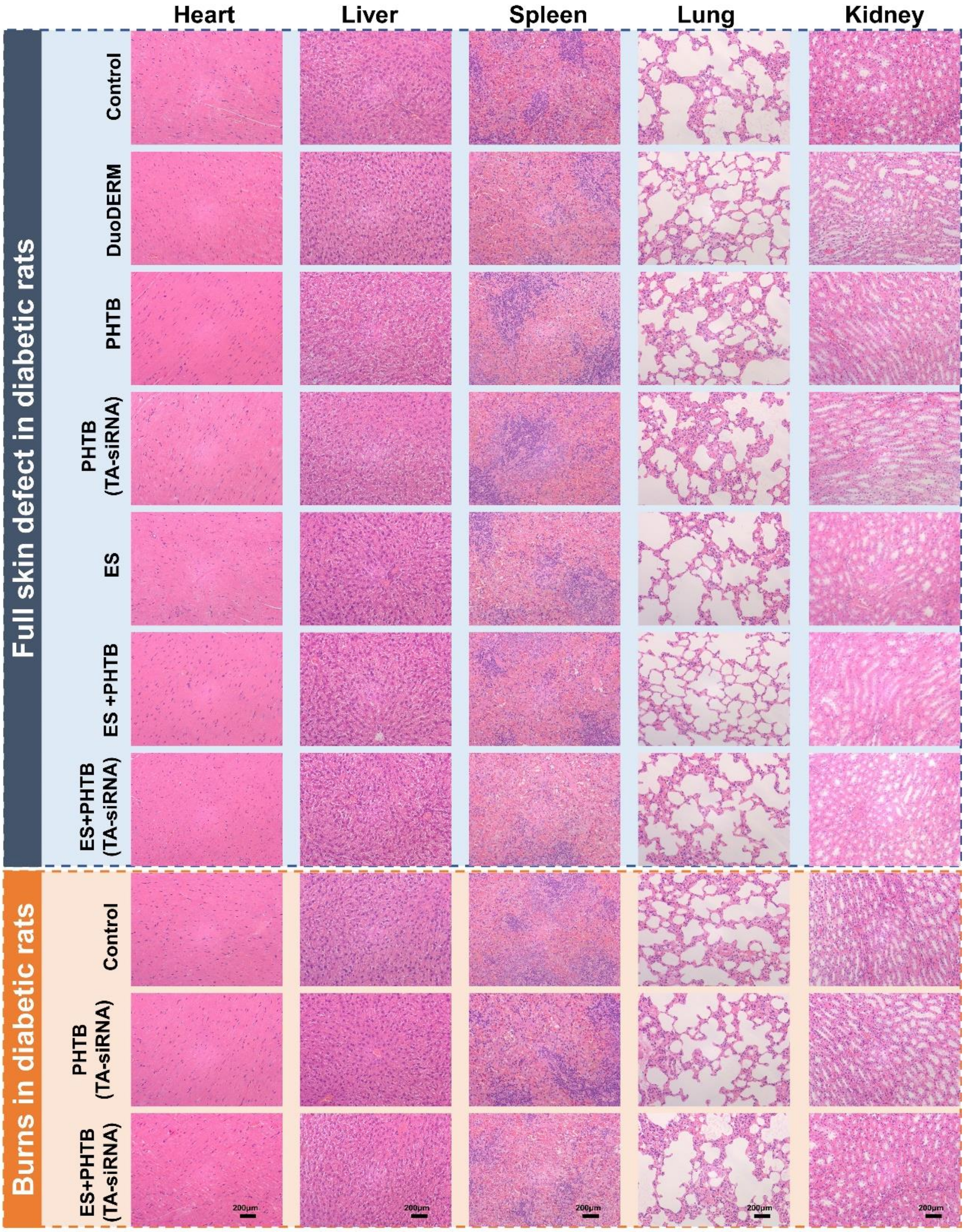

Figure S10. H&E staining of major organs (including heart, liver, kidney, lung and spleen) in diabetic rats at day 7 after full skin defect injury and at day 10 of burn wounds in diabetic rats

Experimental Section

Table S1. siRNA sequences

	Sense (5'-3')	Antisense (5'-3')
siRNA(MMP-9)	CGAGCUAUCCACUCAUCAAdTdT	UUGAUGAGUGGAUAGCUCGGdT
Control (N.C.)	AUCGUACGUACCGUCGUAUdTdT	AUACGACGGUACGUACGAUdTdT
5'FAM-siRNA (MMP-9)	CGAGCUAUCCACUCAUCAAdTdT	UUGAUGAGUGGAUAGCUCGGdT
5'FAM-Control (N.C.)	AUCGUACGUACCGUCGUAUdTdT	AUACGACGGUACGUACGAUdTdT

Characterization of self-assembled TA-siRNA nanogels: The nanogel morphology was observed by scanning electron microscopy (SEM) and transmission electron microscopy (TEM) as follows: the prepared TA-siRNA nanogel reserve solution was dropped on a silicon wafer, placed at room temperature for more than 6 h to ensure water evaporation, and the sample was sprayed with gold by ion sputtering and observed by SEM; the prepared TA-siRNA nanogel reserve solution was dropped on a The prepared TA-siRNA nanogel reserve solution was dropped on a 200-mesh copper mesh, placed at room temperature for more than 6 h to ensure water evaporation, and observed by TEM. Detection of gel fraction of nanogel: the centrifugally filtered nanogel was freeze-dried and weighed recorded as W1, and the centrifugally filtered liquid was collected and freeze-dried and weighed W2, and the gel fraction was calculated by Equation 1.

$$Gel\ fraction = \frac{W_1}{W_1+W_2} \times 100\% \qquad (1)$$

Characterization of hydrogels: The dynamic rheological behavior of hydrogels was analyzed using a rheometer at 25 °C with a test gel volume of 0.2 mL. The tests were performed in the following modes. (I) time-scan test at a constant frequency of 1 Hz and strain of 1%, measuring the storage modulus (G′) and loss modulus (G″). (II) Time scan with constant frequency of 1 Hz and alternating step strain switching from 1% to 200% to measure the storage modulus (G′) and loss modulus (G″) of the hydrogel. (III) To determine the critical strain region of the hydrogel, a strain scan was performed in oscillation mode from 0.01 to 1000% at ω=10 rad/s, and the storage modulus (G′) and loss modulus (G″) were measured. (IV) Frequency scans were performed in the range of 0.1-100 rad/s at 1% strain amplitude to measure the storage modulus (G′) and loss modulus (G″). Conductivity: PHTB hydrogels and PHTB(TA-siRNA) hydrogels were stretched to different lengths (3 cm, 9 cm, 15 cm, 21 cm, 27 cm) and the resistance values were measured by a multi-channel touch screen digital meter(DMM6500, Tektronix). The conductivity (σ) of the hydrogel was measured by using a rectangular mold of 50 mm (length) × 10 mm (width) × 0.25 mm (thickness), with the hydrogel filled in the mold, copper sheets placed on both sides of the mold, and two wires connected to a multi-channel touch screen digital meter to detect the resistance values. The conductivity was calculated as shown in Equation 2, where L is the length and S is the contact area between the hydrogel and the copper sheet.

$$\sigma = \frac{L}{RS} \tag{2}$$

Anti-oxidation properties: DPPH scavenging assay: DPPH (3.0 ml, 100 μM) and dispersion of samples (1 mg) in methanol was stirred and incubated in a dark place for

15 min. The mixture was stirred and allowed to stand in the dark for 15 min. absorbance values at 517 nm were measured by UV-Vis spectrophotometer. the clearance of DPPH was calculated according to equation 3, where A_{blank}, $A_{hydrogel}$ are the absorbance values of blank (DPPH/ethanol solution) and hydrogel (DPPH/ethanol + hydrogel) at 517 nm, respectively. ABTS radical cation (ABTS·+) scavenging assay: ABTS·+ was produced by the reaction of 7.4 mM ABTS solution with 2.6 mM potassium persulfate ($K_2S_2O_8$), and the mixture needed to be placed in the dark at room temperature for 24 h. Before use, the ABTS·+ solution was adjusted with ultrapure water to an absorbance at 734 nm of 0.70 ± 0.02. Then, the PHTB (TA-siRNA) adaptive hydrogel samples (10 mg) were mixed with 3 mL of ABTS·+ solution and incubated in the dark for 20 min. the absorbance of the solution at 734 nm was measured. the ABTS·+ scavenging effect was calculated according to equation 4, where A_{blank}, $A_{hydrogel}$ are the absorbance of ABTS·+ solution at 734 nm before and after mixing with the sample, respectively. H_2O_2 scavenging assay: PHTB (TA-siRNA) adaptive hydrogel hydrogel (1 g) was mixed with H_2O_2 (1 mM, 10 mL) and placed at 37°C. The supernatant was collected at different time points, and the supernatant (50 μL) was mixed with 100 μL of Ti(SO4)2 solution (1.33 mL of 24% $Ti(SO_4)_2$ solution and 8.33 mL of H_2SO_4 mixed with 50 mL of deionized water), and the absorbance at 405 nm was read after 30 min.

$$\text{DPPH scavenging (\%)} = \left(\frac{A_{blank} - A_{hydrogel}}{A_{blank}}\right) \times 100 \quad (3)$$

$$\text{ABTS}^{\cdot+} \text{ scavenging (\%)} = \left(\frac{A_{blank} - A_{\ hydrogel}}{A_{blank}}\right) \times 100 \quad (4)$$

Diabetes rat model: The clean-grade mature male SD rats (200-250 g) were fasted overnight and injected intraperitoneally with citrate buffered 1% streptozotocin solution

at 65 mg/kg. 3 days later, blood was collected from the tail vein of rats for blood glucose testing, and blood glucose greater than or equal to 16.7 mmol/L was considered as a successful model of diabetes. The fasting blood glucose of the rats was tested every 1 day to ensure that the blood glucose was stable above 16.7 mmol/L. When the blood glucose dropped, the rats were injected intraperitoneally with 1% streptozotocin solution in citrate buffer again. After 2 weeks, the rats were randomly divided into 2 groups to establish the diabetic rat full skin defect model and the diabetic rat burn model, respectively.

PHTB(TA-siRNA)-adaptive hydrogel combined with ES promotes full skin defect wound repair in diabetic rats: The rats were injected with anesthetic intraperitoneally, the dorsal hair was removed, and the skin area was disinfected with 75% alcohol, then the full-thickness skin wounds (8 mm in diameter) were cut from the back of each rat by punch biopsy, the hydrogel and the DuoDERM dressing were applied to the skin wounds, respectively. Then, each rat wound was covered with gauze and polyurethane film for fixation. The wounds in the blank control group were covered by sterile gauze and polyurethane film, and the SD rats in each group were observed daily after surgery to ensure that the wounds were always effectively covered. Starting from the second day, the rats in the ES group received pulsed ES daily while ensuring that the hydrogel covered the wounds. The wounds of each group of rats were carefully observed and photographed, and the wound area was calculated by Photoshop software to draw a wound healing trajectory. The wound healing rate was calculated according to Equation 6, where "Area0" represents the initial wound area and "Arean" represents the wound

area at different times of repair.

$$\text{Wound Healing}(\%) = \frac{Area_0 - Area_n}{Area_0} \times 100\% \qquad (6)$$

PHTB(TA-siRNA)-adaptive hydrogel combined with ES promotes burn wound repair in diabetic rats: A deep second-degree burn rat model was established by a tabletop temperature-controlled scalding instrument (YSL-5Q, Jinan Yiyan Technology Development Co., China). The procedures for assessing the repair of the burns of the diabetic rats to which the combination of ES therapy and PHTB(TA-siRNA) hydrogels was applied are shown in Supplementary Figure S11.SD rats were anesthetized and then debrided on the back. To create a third-degree burn wound, a flat-ended aluminum metal rod (1.5 cm in diameter) was electrically heated to 100°C and 10 s of contact with the back skin of the rats allowed the establishment of a deep second-degree burn. After 2 days of burns, the crusted wounds were debrided to remove necrotic tissue. The hydrogel and the DuoDERM dressing were applied to the skin wounds, respectively. Then, each rat wound was covered with gauze and polyurethane film for fixation. The blank group wounds were only covered by sterile gauze and polyurethane film, and the SD rats in each group were observed daily after surgery to ensure that the wounds were always effectively covered. Starting from the next day, the rats in the ES group received pulsed ES daily while ensuring that the hydrogel covered the wounds. The pulsed current was applied at a frequency of 1 Hz and an intensity of 8 mA for 30 min for 20 consecutive days; polarity switching was performed on day 10. The wounds of each group of rats were photographed and recorded and the wound area was calculated by Photoshop software, and the wound healing trajectory was plotted, as well as the wound

healing rate was calculated according to Equation 6.

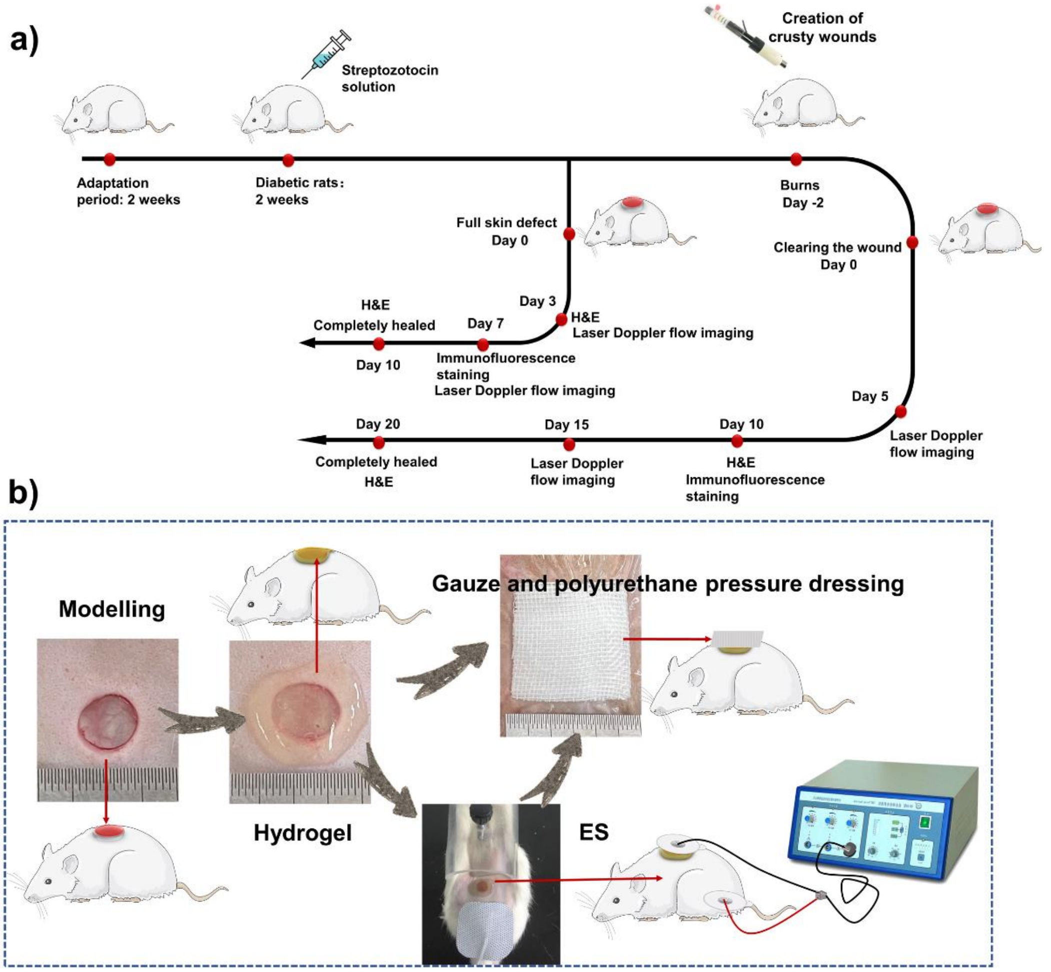

Figure S11. a) Diabetic rat wound modeling time planning diagram b) Schematic diagram of wound treatment and hydrogel combined with ES in rats
